# Cytotoxic Synurins A–C: Chlorinated Naphthoquinone Pigments from the Freshwater Alga *Synura sphagnicola*

**DOI:** 10.1021/acs.jnatprod.6c00417

**Published:** 2026-05-26

**Authors:** Magda Škaloudová, Jan Blahut, Jan Hájek, Alan Kádek, Peter Mojzeš, Jana Pilátová, Dominika Tučková, Petra Divoká, Antonín Střížek, Martin Lukeš, Eva Kotabová, Petra Bittnerová, Kumar Saurav, Pavel Hrouzek, Petra Urajová

**Affiliations:** † Faculty of Science, Charles University, Prague 128 43, Czech Republic; ‡ Institute of Organic Chemistry and Biochemistry, 89220 Czech Academy of Sciences, Prague 160 00, Czech Republic; § Centre Algatech, 86863 Institute of Microbiology of the Czech Academy of Sciences, Třeboň 379 01, Czech Republic; ∥ BIOCEV, Institute of Microbiology of the Czech Academy of Sciences, Vestec 252 50, Czech Republic; ⊥ Institute of Physics, Faculty of Mathematics and Physics, Charles University, Prague 121 16, Czech Republic; # Institute of Parasitology, Biology Centre, Czech Academy of Science, České Budějovice 370 05, Czech Republic; ∇ Faculty of Science, University of South Bohemia, České Budějovice 370 05, Czech Republic; ○ Faculty of Agriculture and Technology, University of South Bohemia, České Budějovice 370 05, Czech Republic

## Abstract

*Synura sphagnicola*, a peat bog chrysophyte, forms conspicuous red cytoplasmic droplets. These droplets previously showed an unidentified pigment, prompting a detailed chemical investigation. Large-scale cultivation and chromatographic purification yielded three structurally related orange-to-red compounds, previously undescribed chlorinated naphthoquinone pigments—synurin A (**1**), synurin B (**2**), and synurin C (**3**)—whose identities were established using HPLC-HRMS, FT-ICR MS, NMR spectroscopy, FTIR, fluorescence spectroscopy, and Raman microscopy. The compounds were identified as 2,3-dichloro-5,8-dihydroxy-6-propylnaphthalene-1,4-dione (**1**) and its two O-methylated regioisomers (**2**, **3**). Raman spectra revealed strong environment-dependent vibrational differences, particularly for **1** and **2**. *In vitro* assays demonstrated potent but nonselective cytotoxicity toward cancerous and nontransformed cell lines (IC_50_ in the low micromolar range), while only **1** exhibited ferric-reducing antioxidant activity.

## Graphical abstract

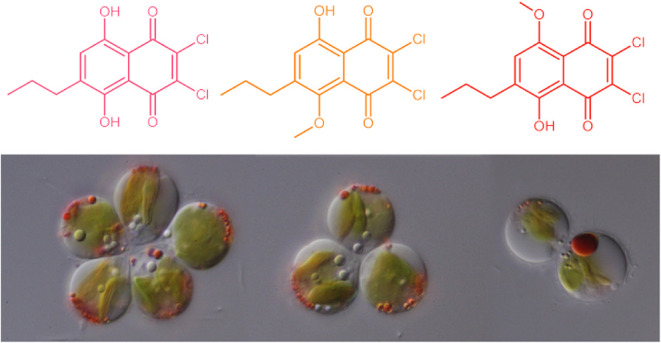

Algae have proven to be a rich source of novel biologically active metabolites with potential biomedical and nutraceutical applications. Various pigments are among the important metabolites of algae.^1–3^ A well-known example is the deep-red carotenoid astaxanthin, a tetraterpenoid produced by green microalga *Haematococcus,* used for its antioxidant, anti-inflammatory, and cardioprotective effects.^4^ Another carotenoid with broad biological activity is fucoxanthin, the main pigment in Ochrophyta (Stramenopiles) algae.^5,6^ The chemical diversity and biosynthetic potential of microalgal pigments continue to expand. Recent discoveries include chrysophaentines, a new structural class of pigments isolated from the chrysophyte *Chrysophaeum taylorii* ^7,8^ and marennine or marennine-like blue pigments found in marine pennate diatoms of the genus *Haslea.* ^9^

Within this broader context of algal pigment diversity, we report previously undescribed pigments from freshwater golden algae of the phylum Ochrophyta, specifically from the class Chrysophyceae, whose characteristic coloration is attributed to high fucoxanthin content.^10^ Our study focuses on the conspicuous red droplets accumulating in the cytoplasm of *Synura sphagnicola* (Synurales, Chrysophyceae), previously shown to contain an unidentified pigment.^11,12^ To date, three additional *Synura* species *S. curtispina*, *S. rubra*, and *S. synuroidea* have been reported to occasionally or consistently form similar red cytoplasmic droplets.^12–15^ All of these species inhabit oligotrophic to mesotrophic humic peat bogs. Historically, such red droplets in *Synura* species were collectively described as accumulations of “haematochrome”,^16,17^ a broad term encompassing various algal carotenoid pigments. In *S. sphagnicola*, high-performance liquid chromatography coupled with high-resolution mass spectrometry (HPLC-HRMS) previously revealed the presence of several pigments, including chlorophyll c_2_, fucoxanthin, zeaxanthin, neoxanthin, and red pigments of unknown composition and structure.^12^

In the present study, cultivation under continuous light regime and large-scale pigment isolation enabled the identification of a new group of chlorinated naphthoquinone-based pigments, designated synurins A–C (**1–3**), named after the producing organism *S. sphagnicola*. Structural elucidation was achieved through a combination of spectroscopic techniques, including high performance liquid chromatography–high resolution mass spectrometry (HPLC-HRMS), ultrahigh resolution Fourier transform ion cyclotron resonance (FT-ICR) MS, nuclear magnetic resonance (NMR), fluorescence and Fourier transform infrared (FTIR) spectroscopy, as well as laser scanning fluorescence confocal microscopy and Raman microspectroscopy. Furthermore, we report *in vitro* cytotoxicity and antioxidant activity for compounds **1–3**.

## Results and Discussion

The purification process of the acetone extract of laboratory-cultivated *S. sphagnicola* yielded three structurally related orange to red compounds, synurin A (**1**), synurin B (**2**) and synurin C (**3**) (Scheme 1). The cultures were not axenic, bacteria were present in the medium. However, the distinct red pigmentation observed within intracellular droplets of *S. sphagnicola* under light microscopy allows us to exclude surrounding bacteria as the source of these pigments. The intracellular synurin content closely correlated with the abundance of droplets: cultures that developed only a few droplets such as those grown under a light–dark regime yielded correspondingly low amounts of synurins.

**Scheme 1:**
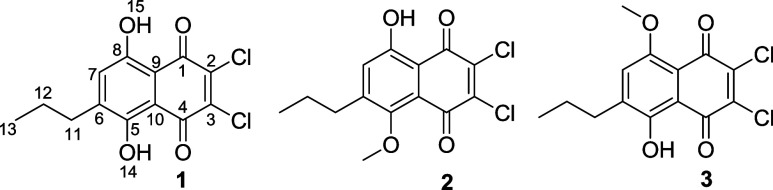
Chemical Structures of Synurins A–C (**1–3**)

Synurin A (**1**, 2.6 mg) was isolated as a red powder. High-resolution mass spectrometric (HRMS-ESI-qTOF) data for **1** showed a protonated molecule at *m*/*z* 301.0034 [M + H]^+^, corresponding to the molecular formula of C_13_H_9_Cl_2_O_4_ with eight degrees of unsaturation (Figure S1). Collision-induced dissociation experiments performed at 30 eV were used to further investigate the structure (Figure S2, compound **1**). Compound **1** generated characteristic fragment ions at *m*/*z* 282.9929 and 264.9801, consistent with sequential losses of two water molecules (C_13_H_9_Cl_2_O_3_^+^, −2.2 ppm and C_13_H_7_Cl_2_O_2_^+^, −0.2 ppm, respectively). Additional fragment ions at *m*/*z* 272.9717 and 259.9639 were attributed to cleavage of the propyl side chain, affording ions with formulas C_11_H_7_Cl_2_O_4_^+^ (−0.4 ppm) and C_10_H_6_Cl_2_O_4_^+^ (−0.4 ppm). Fragmentation involving HCl elimination was also observed, producing ions at *m*/*z* 265.0260 (C_13_H_10_ClO_4_^+^, + 0.8 ppm) and 229.0494 (C_13_H_9_O_4_^+^, + 0.7 ppm). Based on ^1^H shift (Figure S3) we identified two ^1^H in a strong intramolecular hydrogen bond (12.95 and 12.60 ppm, H-14 and H-15), one CH_3_ group (1.01 ppm, H-13), two CH_2_ group with low chemical shift (i.e., not bounded to O) at 2.68 and 1.68 ppm (H-11 and H-12) and one aromatic ^1^H (7.08 ppm, H-1). The COSY experiment (Figure S4), as well as HSQC (Figure S5) combined with HMBC (Figure S6), indicates that the observed aliphatic signals at 2.68, 1.68, and 1.01 ppm correspond to H-11, H-12 and H-13, respectively, forming propyl group. Based on HSQC all four hydrogen-bearing ^13^C were assigned. The chemical shifts indicate that all nine non-hydrogen-bearing carbons are members of aromatic rings where four of them have δ_13C_ > 165 ppm, indicating they are bound with oxygen (Figure S7). Having ten aromatic carbons with two very strong hydrogen bonds and 4 oxygen atoms, a derivative of 5,8-dihydroxy-1,4-naphthoquinone is the only reasonable explanation. Atoms C-6 and C-7 were distinguished from C-3 and C-2 using H-14/C-6 and H-15/C-7 HMBC contacts with H-14 and H-15, respectively. Further, as we observe the HMBC correlation H-7/C-11 as well as between H-11/C-7, the propyl group must be localized at position 6, and therefore, chlorine atoms occupy positions 2 and 3. Observed shifts and *J* interaction are listed in Table 1, all observed COSY, HSQC and HMBC correlations are listed in Table S1 as well.

**Table 1:** ^1^H and ^1^C NMR Spectroscopic Data (500 MHz, CDCl_3_) for Compounds **1–3**

	1		2		3	
Position	δC, type	δH (*J* in Hz)	δC, type	δH (*J* in Hz)	δC, type	δH (*J* in Hz)
1	171.4, C	-	180.7, C	-	181.8, C	-
2	140.8, C	-	141.7, C	-	146.2, C	-
3	140.3, C	-	145.1, C	-	140.6, C	-
4	172.3, C	-	174.1, C	-	173.1, C	-
5	167.4, C	-	155.2, C	-	156.9, C	-
6	148.8, C	-	151.2, C	-	143.8, C	-
7	130.9, CH	7.08 t (0.9)	127.0, CH	7.19 s	123.4, C	7.24 s
8	167.6, C	-	159.9, C	-	155.6, C	-
9	109.5, C	-	111.9, C	-	113.9, C	-
10	110.2, C	-	120.8, C	-	112.9, C	-
11	32.0, CH_2_	2.68 td (7.6, 0.9)	32.6, CH_2_	2.69 t (7.5)	32.7, CH_2_	2.75 t (7.5)
12	21.9, CH_2_	1.68 tq (7.6, 7.3)	23.0, CH_2_	1.67 tq (7.5, 7.5)	22.4, CH_2_	1.70 tq (7.5, 7.3)
13	14.0, CH_3_	1.01 t (7.6)	14.1, CH_3_	1.00 t (7.5)	14.1, CH_3_	1.01 t (7.3)
14	-	12.95 s	62.0, CH_3_	3.85 s	—	12.72 s
15	-	12.60 s	—	12.26 s	57.0, CH_3_	4.01 s

To further support the proposed structure, experimental (FTIR) spectra were compared with vibrational spectra calculated at the DFT (density function theory) level using Gaussian calculations. Only moderate agreement was achieved, primarily due to the low signal-to-noise (S/N) ratio and the presence of impurities affecting the recorded infrared data (Figure S8, compound **1**). While hydroxy group stretching vibrations are not clearly detectable, bending vibrations in the **1410–1440 cm** ^**–1**^ region **(R3)** and a strong absorption near **1200 cm** ^**–1**^ **(R1)** are consistent with the absence of O-methylation in both hydroxy positions. The presence of two chlorine atoms strongly influences vibration in **R4** region (**mainly 1540–1600 cm** ^**–1**^), as the calculated spectra shows prominent C=C stretching of chlorinated carbon; and **R5 (1620–1700 cm** ^**–1**^) with important the C=O stretching of neighboring carbonyl.

Synurin B (**2**, 1.09 mg) was isolated as an orange powder. FT-ICR mass spectrometry was used to directly determine its molecular formula and to confirm the presence of chlorine incorporated in the molecule through isotopic fine structure fitting (Figure S9). The predominant ion corresponded to the sodium adduct [M + Na]^+^ at *m*/*z* 337.00050, consistent with the molecular formula C_14_H_12_Cl_2_NaO_4_^+^ (mass error: −0.06 ppm). A dimeric sodium adduct [2M+H+Na]^+^ was also detected at *m*/*z* 653.01249, supporting the formula C_28_H_24_Cl_4_NaO_8_^+^ (−1.14 ppm). The protonated molecule [M + H]^+^ was observed at lower relative abundance at *m*/*z* 315.01856, corresponding to the molecular formula C_14_H_13_Cl_2_O_4_^+^ (mass error: −0.07 ppm). These results are consistent with the initial ESI-qTOF data, in which **2** was detected as the [M + H]^+^ ion at *m*/*z* 315.0197 (mass error: −3.5 ppm) (Figure S1). Fragmentation experiments performed on the HRMS-ESI-qTOF instrument revealed a less complex fragmentation pattern compared to **1** (Figure S2, compound **2**). Major ions were observed at *m*/*z* 297.0092 and 264.9812, corresponding to loss of water (C_14_H_11_Cl_2_O_3_, −0.7 ppm) followed by loss of a methoxy group (C_13_H_7_Cl_2_O_2_^+^, +2.1 ppm), respectively. Direct loss of the methoxy group from the protonated molecules yielded a fragment at *m*/*z* 282.9920 (C_13_H_9_Cl_2_O_3_^+^, +1.3 ppm). Additional ions resulting from side-chain cleavage were detected at *m*/*z* 286.9866 and 271.9640 (C_12_H_9_Cl_2_O_4_^+^, +2.3 ppm and C_11_H_6_Cl_2_O_4_^+^, +8.3 ppm, respectively). Minor peaks arising from HCl elimination were observed at *m*/*z* 279.0421 (C_14_H_12_ClO_4_^+^, −0.9 ppm) and 243.0650 (C_14_H_11_O_4_^+^, +0.7 ppm). Compound **2** provides very similar ^1^H, ^13^C, HSQC and HMBC (Figures S10–S13) spectra as compound **1**, but with an additional CH_3_ group and only one hydrogen-bonded ^1^H with δ_1H_ > 12 ppm. According to the chemical shift of (3.85 ppm), the CH_3_ signal corresponds to the methoxy group. Strong HMBC (Figure S13) correlation was detected between this group (H-14) and C-5 which localizes the methoxy group to the ortho position with respect to the propyl group. This is further proved by HMBC correlations H-15/C-8 and H-15/C-7 localizing the OH group on the other side of the naphthoquinone ring. Observed shifts and *J* interaction are listed in Table 1.

The experimental FTIR spectrum of **2** exhibited good quality (S/*N* > 8), enabling reliable assignment of absorption bands. After normalization, the calculated spectra showed excellent agreement with the experimental data (Figure S8, compound **2**). The presence of two chlorine substituents strongly influenced the C=C stretching vibrations, giving rise to an intense absorption band around **1550 cm** ^**–1**^ **(R4)**. The carbonyl C=O stretching vibration, affected by the neighboring chlorine atoms, appeared at **1655 cm** ^**–1**^ **(R5)**. Together with the O–H bending vibration at **1530 cm** ^**–1**^ **(R4)**, these bands showed close correspondence between calculated and experimental spectra, supporting the proposed structure. Vibrations of the aliphatic side chain were observed in the **1300–1440 cm** ^**–1**^ region **(R3)**, while methoxy group bending vibrations appeared around **1280 cm** ^**–1**^ **(R2)**. Aromatic C–H bending modes were detected at **1170 cm** ^**–1**^ and **1220 cm** ^**–1**^ **(R1)**. The best spectral agreement was obtained for the dimeric form, stabilized by hydrogen bonding between aromatic hydroxy groups. Although this dimerization significantly affects the IR spectra, particularly in the **1300–1500 cm** ^**–1**^ region **(R3)**, the spectral correspondence alone cannot be considered conclusive evidence for dimer formation. Due to presence of methoxylation, absorption in **R4** and **R5** show lower intensity than corresponding signals in **1**. The tentative O–H stretching band was observed in the range of 2850–3000 cm^–1^, which is significantly lower than typically expected. This may be attributed to the extensive formation of strong hydrogen bonds, which consequently leads to a poor agreement between the calculated and experimental spectra in this region.

Synurin C (**3**, 0.8 mg) was isolated as a red powder. HRMS-ESI-qTOF analysis revealed a protonated molecule at *m*/*z* 315.0197 [M + H]^+^, consistent with a molecular formula of C_14_H_12_Cl_2_O_4_ and eight degrees of unsaturation (Figure S1). Its fragmentation pattern closely resembled that of **2** (Figure S2, compound **3**), indicating that compounds **2** and **3** are regioisomers differing in the position of the methoxy group relative to the propyl substituent. Similarly, the NMR spectra (Figures S14–S18) of **3** closely resemble those of **2**, i.e., a single ^1^H signal of H-bounded OH and a single signal of the methoxy group. The methoxy group is, however, localized on the other side of the naphthoquinone ring (meta with respect to the propyl) which was proved by HMBC, where we see correlation between H-15/C-8 and corelation between H-14 and carbons C-5, C-6 and C-10 but not with C-9 and C-8 (Figure S15). Additionally, NOE contact was observed between H-15/H-7 only for **3** but not for **2** under the same conditions (Figure S19). Observed shifts and *J* interaction are listed in Table 1.

The overall FTIR spectral profile of **3** was similar to that of **2** (Figure S8, compound **3**). The low concentration of the compound does not allow clear overlay, as the S/N ratio remains low and some minor impurities stays in the compound. Clear identification of the compound is again supported by intense aromatic C=C/CO stretching in region **R4** and **R5** almost identical to those reported in **2**. However, vibrations associated with the alkyl side chain and aromatic C–H were additionally observed around **1190 cm** ^**–1**^ **(R1)**, reflecting the altered spatial relationship between the methoxy group and the propyl chain. Region **R2** and **R3** are again highly similar to those observed in **2**, as these regions shows vibration of methoxy- and aliphatic side chains without any effect on the cyclic part.

Based on HRMS and NMR information, it can be concluded that **1–3** were identified as 2,3-dichloro-5,8-dihydroxy-6-propylnaphthalene-1,4-dione (**1**–synurin A), 2,3-dichloro-8-hydroxy-5-O-methyl-6-propylnaphthalene-1,4-dione (**2**–synurin B) and 2,3-dichloro-5-hydroxy-8-O-methyl-6-propylnaphthalene-1,4-dione (**3**–synurin C), respectively.

All these orange/red pigments dissolved in MeOH exhibit three main absorption maxima in UVC (220 nm), UVB (290–300 nm), and the visible range (∼470–510 nm). Autofluorescence of red droplets in *S. sphagnicola* cells has previously been reported, with a broad emission maximum around 630 nm upon excitation at 442 nm.^12^ We measured fluorescence spectra of the pure pigments dissolved in MeOH using excitation wavelengths of 225, 300, and 500 nm. Almost no or very weak fluorescence was detected with excitation at 225 and 500 nm (data not shown), whereas excitation at 300 nm produced the most distinct emission spectra (Figure 1).

**Figure 1:**
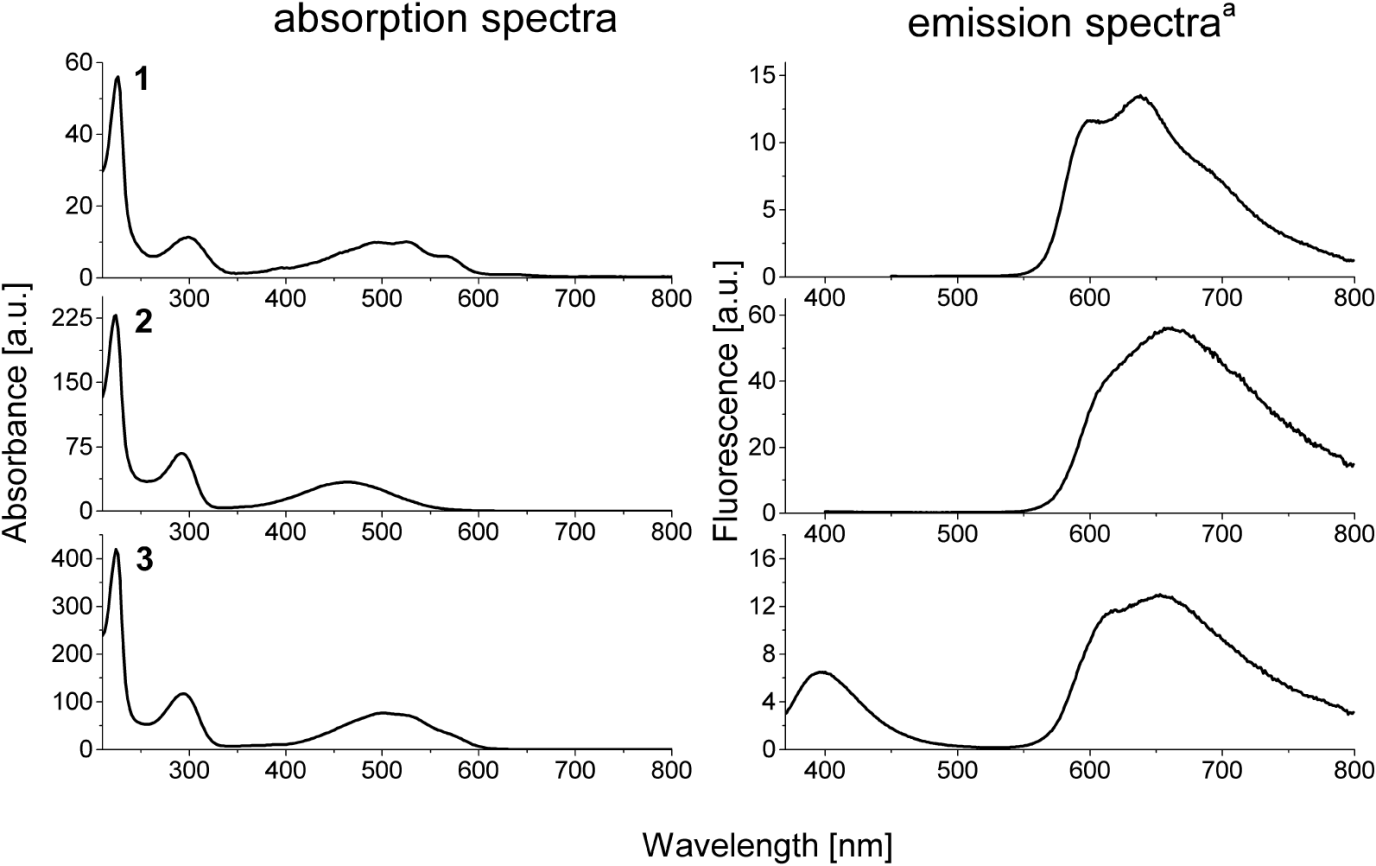
Absorption and emission spectra of compounds **1–3**. ^a^Fluorescence emission spectra were measured with excitation at 300 nm.

Compound **1** showed maximum emission at around 640 nm, while **2** and **3** exhibited maxima at around 660 nm. Interestingly, **3** also displayed a local emission maximum at 400 nm. As this feature was observed only in this compound, we suggest it may result from the spatial orientation of the propyl chain relative to the methoxy group. The emission maxima of pure compounds are generally red-shifted relative to the autofluorescence observed in intact cells, which might reflect differences in local environments or interactions with other cellular components. The excitation-dependent behavior, particularly the strong response at 300 nm, suggests that **1–3** may participate in UVB-light sensing or energy dissipation. Moreover, the compound-specific shift in emission wavelength suggests that small structural variations can modulate photophysical properties, potentially affecting pigment function *in vivo*. These observations warrant further investigation into the role of **1–3** in photoprotection or light harvesting in *Synura* species.

### Confocal Laser Scanning Microscopy

To further investigate the intracellular localization of **1–3**, living *S. sphagnicola* cultures were examined using confocal laser scanning microscopy (Figure 2).

**Figure 2:**
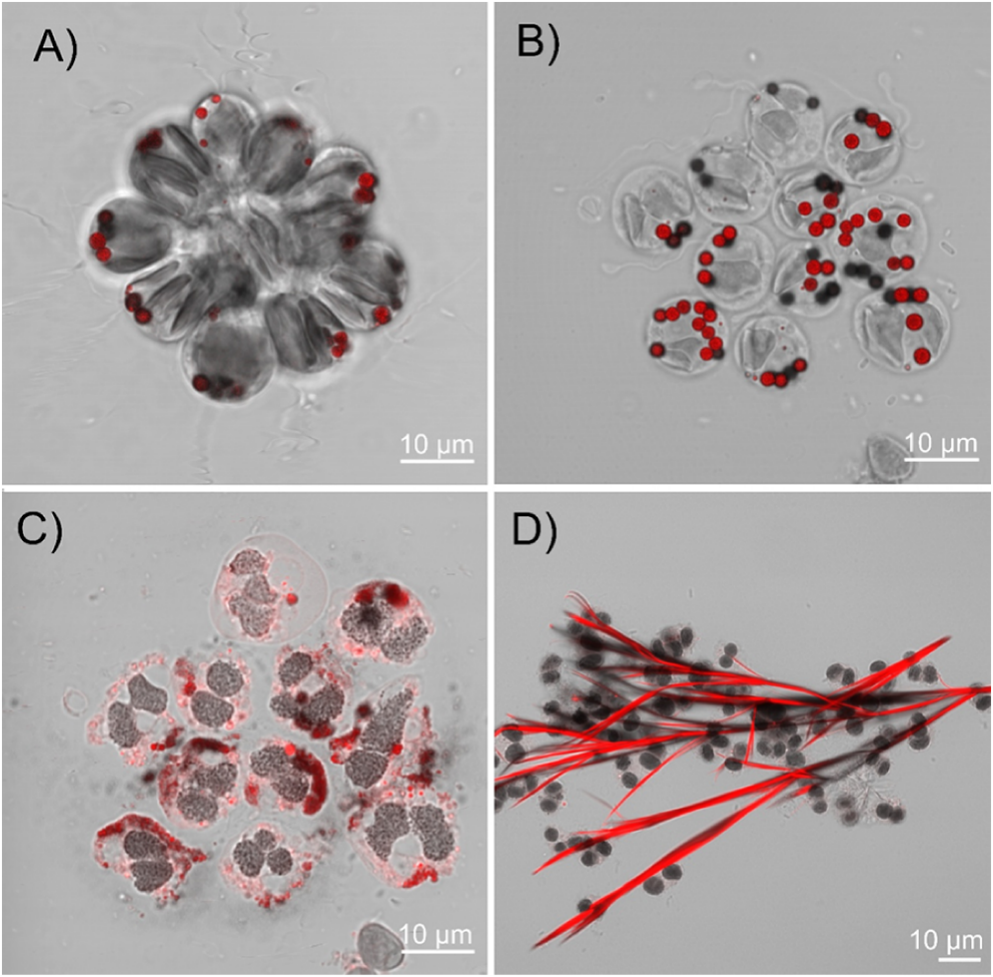
Confocal microscopy of *S. sphagnicola* colony. A) Intact colony containing intracellular pigment droplets. B) Colony disintegrated into individual cells. C) Progressive disruption of the cellular membrane accompanied by the release of red pigment into the surrounding environment. D) Formation of needlelike pigment crystals from the released pigment following cell death.

Red pigments were excited using a 543 nm laser and fluorescence emission was detected in the range 570–627 nm to avoid overlap with chlorophyll autofluorescence. The images were taken using transmitted light to provide a clearer view of the distribution of the pigment within the cell and its surroundings.

In intact colonies (Figure 2A), red pigment droplets were predominantly localized at the colony periphery. Following cell death, the colony rapidly disintegrated into individual cells (Figure 2B), and progressive membrane disruption was accompanied by the gradual release of red pigment into the surrounding environment (Figure 2C). Cells that had been dead for an extended period exhibited crystalline pigment aggregates in the extracellular space, consistent with the low solubility of these compounds (Figure 2D). Collectively, these observations indicate that droplets containing compounds **1–3** remain confined within intact cells and are released only upon membrane rupture, supporting a natural intracellular role for these metabolites. The subsequent extracellular crystallization underscores their hydrophobic character and likely reflects a passive, postmortem accumulation process rather than active secretion. Such behavior may carry ecological significance, potentially contributing to allelopathic interactions or defensive mechanisms following cell lysis.

### Raman Microspectroscopy

The Raman spectra of **2** and **3** dissolved in MeOH are practically identical, except for minor differences in the relative intensities of some Raman bands (Figure 3). This high degree of spectral similarity reflects the structural similarity of the two compounds, which are regioisomers differing only in the ortho and para positions of the methoxy group relative to the propyl group for **2** and **3**, respectively. On the other hand, the spectrum of **1** dissolved in MeOH differs significantly in the positions of some intense Raman bands, which seem to be shifted counterparts of the bands observed in the spectra of **2** and **3**. For example, the bands at 448, 1193, and 1418 cm^–1^ present in the spectrum of **1** can be considered as shifted Raman bands of **2** and **3**, centered at 472, 1207, and 1387 cm^–1^, respectively. These frequency shifts are likely a consequence of replacing the methoxy group in **2** with a hydroxy group in **1** but may also reflect intramolecular hydrogen bonding and the coexistence of tautomeric forms, which can be expected for **1** and **2**.

**Figure 3:**
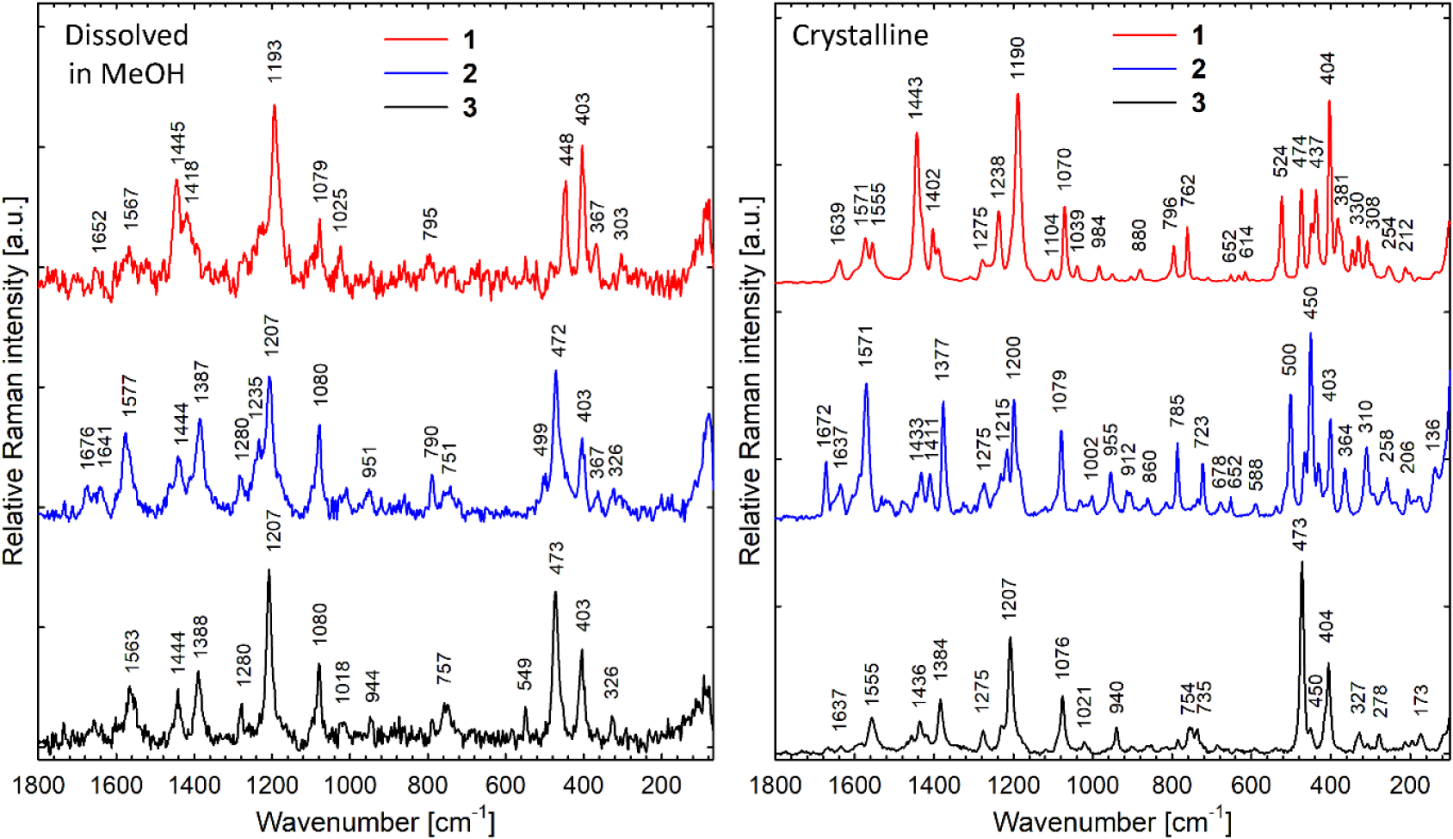
Raman spectra of compounds **1–3** obtained from their MeOH solutions and from crystals formed after evaporation of MeOH. For detailed crystal structure of compounds **1–3** see Figures S20, S21, S22. In both cases, the spectra were excited at 785 nm. The spectral contribution of MeOH was carefully subtracted.

The influence of the environment, the coexistence of tautomeric forms, and the formation of intra- and intermolecular hydrogen bonds undoubtedly play a significant role in the vibrational states of synurins, as the transition from the dissolved state to the crystalline state is accompanied by dramatic changes in their Raman spectra. As can be seen from the comparison of the spectra of these compounds dissolved in MeOH and the spectra of their crystalline forms (compare spectra in both panels shown in Figure 3), only the Raman spectra of **3** remain practically the same after crystal formation, except for some minor spectral shifts and an overall narrowing of the bands, a general consequence associated with a higher degree of molecular ordering in the crystal structure. In contrast, a number of new Raman bands appear for the crystalline form of **2**, which were not present at all (or only as very weak bands) in the spectra of MeOH solutions. In a similar way, the incorporation of molecules into a regular structure of a molecular crystal is also manifested in **1**. The appearance of new, relatively intense Raman bands (e.g., 762, 524, and 474 cm^–1^) may indicate the coexistence of several tautomeric or conformational forms stabilized by intra- or intermolecular interactions, as well as intramolecular hydrogen bonds due to the proximity of carbonyl and hydroxy groups. Only in the case of **3** can it be concluded, based on the exceptional similarity of the spectra in solution and in the molecular crystal, that its molecular structure does not change much upon crystal formation. A more detailed interpretation of the reasons for those spectral differences would require quantum-mechanical calculations of the Raman spectra of molecules embedded in crystal structures, which are not currently available.

### Occurrence of Naphthoquinone (NQ)-Based Pigments and Their Pro/Antioxidant Activity

The synurins are naphthoquinone (NQ)-based pigments that represent a class of redox-active secondary metabolites derived from the naphthalene scaffold, whose physicochemical properties are strongly influenced by the type and position of substituents on the aromatic core. These metabolites are widespread in plants, fungi, and bacteria, and to a lesser extent also in animals (Table S2).^18–22^ Remarkably, they have not been documented in algae, with the sole exception of deoxylapachol reported from the marine brown alga *Landsburgia.* ^23,24^ NQ-based pigments also occur among the secondary metabolites of lichens; however, these compounds are produced exclusively by the fungal partner (mycobiont), and no evidence to date indicates NQ biosynthesis in the algal symbiont (phycobiont).^25,26^

Structurally related natural products provide valuable context for interpreting the biological activity of synurins. Among the most extensively studied compounds with similar structural features are NQ derivatives substituted with various functional groups such as chlorine, ethyl, acetyl, methoxy and hydroxy groups (Table S2). Several of them have been reported to either induce oxidative stress or, conversely, protect from the same through intrinsic or induced antioxidant activity.^20,27–29^ Notably, all 1,4-NQ compounds showing pro/antioxidant activity are substituted with at least one hydroxy group. Here, we evaluated the pro/antioxidant activity of synurins using two complementary assays. However, strong spectral interference of the compounds in the UV–Vis region prevented reliable quantification using the classical DPPH (2,2-diphenyl-1-picrylhydrazyl) assay.^30^ Therefore, to assess the antioxidant potential of compounds **1–3**, we employed the ferric reducing antioxidant power (FRAP) assay,^31^ which quantifies the ability of a compound to reduce ferric ion (Fe^3+^) to ferrous ion (Fe^2+^). Ascorbic acid served as a positive control. Among the tested analogues, only compound **1** exhibited measurable reducing activity, corresponding to 1.249 μg ascorbic acid equivalents (AAE) per μg of compound. These findings indicate that compounds **2** and **3** lack ferric-reducing activity; however, this does not exclude the possibility that they may exert antioxidant effects via alternative mechanisms.

### Cytotoxic Properties, MTT Assay

Natural 1,4-NQ derivatives including juglone, cristazarin, naphthazarin, plumbagin and 3-chloroplumbagin, the latter representing the only naturally occurring chlorinated 1,4-NQ pigment reported to date, are well-known for their cytotoxic properties.^22,25,32,33^ Owing to their redox activity and structural versatility, 1,4-NQ scaffolds are widely employed in both natural and synthetic compounds with antitumor activity. Structurally, **1–3** most closely resemble naphthazarin (5,8-dihydroxy-1,4-naphthoquinone), a metabolite reported to exert multiple biological effects, including inhibition of cell proliferation and induction of apoptosis in human colorectal and gastric cancer cell lines.^34^ Given the structural similarity of synurins to these bioactive 1,4-NQ derivatives, we further examined their cytotoxic properties. Compounds **1–3** were evaluated using the MTT assay^35^ on human colorectal (HCT116) and breast cancer (MDA-MB-231) cell lines, as well as on an immortalized retinal pigment epithelial cell line (hTERT-RPE-1) (Table 2, Figure S23).

**Table 2:** IC_50_ Values (μM) of **1–3** on Different Cell Lines

	Cell lines		
	HCT116	MDA-MB-231	hTERT-RPE-1
**1**	0.28	0.15	0.39
**2**	2.6	2.1	1.7
**3**	1.1	0.82	1.7
Nostatin A	0.020	0.016	0.016

All synurin analogues displayed dose-dependent cytotoxicity, with IC_50_ values in the very low micromolar range, comparable across both cancerous and noncancerous cell types. Their IC_50_ value is similar to that reported for juglone, plumbagin, or 3-chloroplumbagin.^36,37^ Importantly, no preferential cytotoxicity toward tumor cell lines was observed, as the sensitivity of the nontransformed hTERT-RPE-1 cells was within a similar range. From a structural perspective, the position of the propyl substituent does not appear to influence cytotoxic activity, whereas the presence or absence of free hydroxy groups at positions 5 and 8 increases the potency by 1 order of magnitude. These findings indicate a lack of tumor-cell selectivity, suggesting that the isolated compounds **1–3** are not directly suitable candidates for anticancer therapy. Nevertheless, their consistent cytotoxic profile highlights synurins as promising scaffolds for the development of more potent and selective derivatives. In addition, understanding the biological role of these pigments in *Synura* taxa represents an intriguing direction for future research.

## Experimental Section

### General Experimental Procedures

Crude organic extract was fractionated using MPLC Büchi Pure C-815 Flash (Büchi, Switzerland). HPLC-HRMS analyses were carried out on Dionex UltiMate 3000 UHPLC+ (Thermo Scientific) equipped with a diode-array detector connected to an Impact HD high-resolution mass spectrometer (Bruker Daltonics). Ultrahigh resolution mass measurements were performed using a Paracell-equipped 15T SolariX XR Fourier-transform ion cyclotron resonance mass spectrometer (FT-ICR MS; Bruker Daltonics) operated in positive ion mode. NMR spectra were acquired in CDCl_3_ at 25 °C using Bruker AVANCE III HD 500 MHz spectrometer with cryoprobe. Chemical shift was referenced on the residual CDCl_3_ signal for ^1^H (7.26 ppm) and CDCl_3_ signal for ^13^C (77.16 ppm). Fourier transform infrared spectra (FTIR) were obtained with a Nicolet IS10 (Thermo Nicolet) spectrometer equipped with Smart iTR accessory with installed ZnSe ATR crystal, KBr beam splitter and DTGS detector. Omnic software (Nicolet) was used for measurement and data processing. Fluorescence emission spectra were measured at room temperature by Aminco-Bowman Series 2 spectrofluorometer (Thermo Fisher Scientific, USA). Confocal microscopy images were acquired by a laser scanning confocal microscope (Zeiss LSM 880; Carl Zeiss Microscopy GmbH) equipped with a Plan-Apochromatic 63×/1.4 Oil DIC M27 objective. Raman microscopy was performed using a WITec alpha300 RSA confocal Raman microscope (Oxford Instruments–WITec, Germany) equipped with a dry 50× LD EC Epiplan-Neofluar Dic, NA 0.55, working distance 9.1 mm (Zeiss, Germany). Data processing consisted of cosmic ray removal, solvent subtraction, and background correction using WITec Project Six Plus v6.2 software (Oxford Instruments–WITec, Germany).

### Species Collection, Identification, and Cultivation

The freshwater chrysophyte alga *S. sphagnicola* was isolated from a water sample (pH 3.9) collected on 7 June 2017 at Klečové louky National Reserve, Jizerské hory, Czech Republic (50°50′14.2″ N, 15°14′45.8″ E). The phylogenetic position and morphological characterization of the strain are described in detail by Škaloud et al.^12^ The strain is deposited in the Collection of Algae of Charles University in Prague, Czech Republic (CAUP), under the accession number CAUP B 714 (corresponds to strain K35 in Škaloud et al.^12^). Cultures were maintained in DYV MES-buffered liquid medium.^38^ For the extraction of red pigments, *S. sphagnicola* was cultivated in 250 mL Erlenmeyer flasks under continuous illumination of 40–60 μmol m^–2^ s^–1^ at 18–23 °C. Prior to harvesting, cells were examined for the presence of red pigments using an Olympus BX51 light microscope equipped with Nomarski interference contrast. Optical density at 750 nm was measured for harvested cultures using a UV–vis absorption spectrometer, yielding values of 0.04–0.08, corresponding to 10–15 mg/L of dry biomass. Cultures were centrifuged at 4430 × g for 10 min, and the biomass was stored at −18 °C until extraction. The total volume of 15 L was grown for isolating over 4 mg of pure pigment fractions for structural analysis and bioactivity assays.

### Pigment Purification

Thawed *S. sphagnicola* biomass was extracted with HPLC-grade acetone after homogenization with 1 mm glass beads for 5 min, repeated three times. After centrifugation, the supernatants were pooled and evaporated to dryness to obtain 70 mg of organic extract. The organic extract was subjected to MPLC using a normal-phase silica gel (100–200 mesh, 40 μm, 12 g) column and eluted with a gradient solvent system of hexane-chloroform (0% to 100%) for 40 min. Fractions were collected using an automatic fraction collector equipped with tubes having a maximum volume of 25 mL per vial. The compound was monitored using a UV–vis detector (500 nm). Using threshold detection parameters set at 0.05 AU for UV detection, a total of twenty-nine fractions (F1–F29) were obtained. Composition of the fractions was checked by HPLC-HRMS analysis, and those containing red pigments were pooled accordingly, concentrated, and subjected to semipreparative C8 column (Agilent Eclipse XDB C8, 5 μm, 9.4 × 250 mm). The mobile phase consisted of H_2_O (A) and MeOH (B). The flow rate was 4 mL/min, and the gradient was as follows: A/B 20/80 (0 min), 20/80 (in 1 min), 0/100 (in 20 min), 0/100 (in 25 min), and 20/80 (in 27 min). Fractions eluted in 13.7 (**3**), 14.2 (**2**), and 18.7 (**1**) min were collected and evaporated to dryness.

### Mass Spectrometry and Ultrahigh-Resolution Mass Spectrometry Analysis

Extracts and fractions were analyzed using LC-HRMS analysis. Separation of compounds was performed on a reversed-phase C18 column (Phenomenex Luna C18 Polar, 150 × 4.6 mm, 2.6 μm) using H_2_O (A) and MeOH (B) (both containing 0.1% formic acid) as a mobile phase at a flow rate of 0.7 mL/min. The gradient was as follows: A/B 20/80 (0 min), 20/80 (in 1 min), 0/100 (in 20 min), 0/100 (in 25 min), and 20/80 (in 27 min). The HPLC was connected to a high-resolution mass spectrometer with electrospray ionization in positive mode. The following settings were used: drying temperature 250 °C; drying gas flow, 11 L/min; nebulizer gas pressure, 3.5 bar; capillary voltage, 4.0 kV; end plate offset, 500 V. Spectra were collected in the range *m*/*z* 20–2200 with a spectra rate of 2 Hz. The collision energy was set to 30 eV. The mass spectrometer was calibrated with sodium formate clusters at the beginning of each analysis.

Ultrahigh resolution mass measurements were performed using 3 μL/min direct infusion and electrospray ionization (ESI) of **2** dissolved in 60% acetonitrile acidified with 0.1% formic acid. The source parameters were as follows: ESI voltage 3.9 kV, nitrogen was used both as the nebulizing gas (1.0 bar) and the drying gas (4 L/min), while the drying capillary temperature was kept at 200 °C. For the MS1 overview spectrum, ions without quadrupole isolation were accumulated and thermalized in a collision cell with 40% argon flow for 0.05 s before detection over 92–1500 *m*/*z* range with 4 Mpts data sampling (FID transient 0.84 s). Subsequently, ions of singly charged **2** isotopic cluster were isolated in a quadrupolar mass filter (8 *m*/*z* window), accumulated for 0.2 s and measured with 8 Mpts sampling (FID transient 1.68 s) with the resulting spectrum processed in absorption mode (Kilgour apodization with factor = 0.5, Simple_100 baseline correction with double zero-filling) in ftmsProcessing v. 2.2.0 (Bruker Daltonics). Molecular formula of **2** was assigned in both mass-selected and MS1 overview spectra using isotopic fine structure fitting implemented in DataAnalysis 6.1 (Bruker Daltonics). We selected 1+ peak for MS/MS fragmentation of compound **2** (1.8 *m*/*z* quadrupole window at 314.5 *m*/*z*), it was subjected to 17 eV collision-induced dissociation (CID) in the collision cell and accumulated for 1 s prior to analysis with 8 Mpts sampling over 92–500 *m*/*z* mass range (FID transient 1.68 s). In all MS experiments, 32 individual scans were summed, and the measurements were externally calibrated using 0.1% NaTFA clusters.

### Nuclear Magnetic Resonance

Resulting ^1^H, ^13^C­{^1^H}, ^1^H–^13^C HSQC, ^1^H–^13^C HMBC and COSY spectra together with the assignment are depicted in Figures S3-S17. The 1D selective NOESY (Figure S19) was measured using gradient selective echo for methoxy group followed by 300 ms NOESY mixing.

### Synurin A (**1**)

Red amorphous solid; 2.6 mg, 3.7% of crude extract, purity 97% (Figure S24); UV (MeOH) λ_max_ (log ε) 225 nm (4.57), 297 nm (3.91), 524 nm (3.84); IR (ATR) νmax 1420, 1255, 1231, 763 cm^–1^^‑^^1^; ^1^H and ^13^C NMR data see Table 1; HRMS *m*/*z* [M + H]^+^ (calcd for C_13_H_10_Cl_2_O_4_^+^ 301.0029, found 301.0031, Δ + 0.7 ppm).

### Synurin B (**2**)

Orange amorphous solid; 1.09 mg, 1.55% of crude extract, purity 96% (Figure S24); UV (MeOH) λ_max_ (log ε) 223 nm (4.62), 291 nm (4.14), 463 nm (3.83); IR (ATR) νmax 1670, 1632, 1582, 1376, 1216, 1171 cm^–1^; ^1^H and ^13^C NMR data see Table 1; HRMS *m*/*z* [M + H]^+^ (calcd for C_14_H_12_Cl_2_O_4_^+^ 315.0185, found 315.0193, Δ + 2.5 ppm).

### Synurin C (**3**)

Red amorphous solid; 0.8 mg, 1.14% of crude extract, purity 95% (Figure S24); UV (MeOH) λ_max_ (log ε) 224 nm (4.46), 293 nm (3.95), 500 nm (3.75); IR (ATR) νmax 1667, 1600, 1233, 1077, 944, 761 cm^–1^; ^1^H and ^13^C NMR data see Table 1; HRMS *m*/*z* [M + H]^+^ (calcd for C_14_H_12_Cl_2_O_4_^+^ 315.0185, found 315.0188, Δ + 1.0 ppm).

### Fourier-Transform Infrared Spectroscopy (FTIR)

Purified **1–3** were dissolved in 10 μL of DCM/MeOH (1:1, v/v) and 1 μL was deposited onto an ATR crystal via Hamilton syringe. After evaporation of the solvent the absorbance spectra were collected in the spectral range from 525 cm^–1^ to 4000 cm^–1^, at a spectral resolution of 4 cm^–1^; 32 scans were coadded; H_2_O and CO_2_ correction was applied. A Blackman-Harris apodization function was used, with a zero-filling factor of 2. Background was measured the same way as the sample, but only DCM/MeOH (1:1, v/v) was used.

### Fluorescence Spectroscopy

Fluorescence emission spectra were measured using a quartz cuvette and standard instrument geometry. Pure **1–3** were dissolved in 90% MeOH at room temperature. The emission spectra were scanned with 4 nm slit width. The excitation was at 300 nm with 16 nm slit width. The instrument function was corrected by dividing raw emission spectra by the simultaneously recorded signal from the reference diode.

### Confocal Microscopy

About 10 μL of culture was placed onto a high precision microscope cover glass slide. Due to the motility of *S. sphagnicola* cells, we immobilized them by placing several strands of cellulose cotton around a drop of culture medium and gently covering it with a coverslip. As the cotton gradually absorbed water from the drop, cells and colonies migrated closer to the cellulose fibers and remained immobilized. By appropriately adjusting the emission filter settings, we were able to suppress chlorophyll fluorescence and highlight the fluorescence of red droplets containing **1–3**. Given the sensitivity of *S. sphagnicola* cells, we also monitored cell death during observation. Images were immediately acquired by a laser scanning confocal microscope. The signal was detected by a GaAsP photomultiplier in 8-bit mode. Synurins were excited using a 543 nm laser with beam splitters MBS 488/543/633. Fluorescence was detected within 570–627 nm.

### Raman Microscopy

Samples of isolated pigments were measured as saturated solutions in HPLC-grade MeOH, as well as needlelike red crystals recrystallized from MeOH solutions on a quartz slide. For MeOH solutions, the samples were placed in a 1 mm quartz absorption cuvette and, thanks to the extended working distance of the objective and the high confocality of the WITec Raman microscope, Raman spectra were acquired from the sample volume without contributions from the cuvette walls. In the same way, the MeOH spectrum was acquired under identical conditions and then subtracted from the solution spectra. The far-red 785 and 830 nm laser excitations were used, with power ranging from 5 to 30 mW. Both far-red excitations provided virtually identical Raman spectra. The far-red excitations were intentionally used to avoid the high fluorescence background and the rapid photodegradation of the crystals observed with other available visible excitations, e.g., 532, 633, or 647 nm. To further reduce potential photodamage or local overheating, even for the far-red excitations, fast Raman mapping over a selected area (typically 15 × 15 μm) covering variously oriented crystals was used, with a 200 nm scanning step in both directions, and a 0.05 s integration time per voxel. While avoiding interference with the fluorescence signal, we still observed a weak but relatively narrow fluorescence background in synurin B (**2**) crystals for both 785 and 830 nm, with a maximum at 882 nm. No similar fluorescence was observed for MeOH solutions. The *True Component Analysis* tool and averaging over at least five technical replicates were used to improve the signal-to-noise ratio and to suppress polarization-dependent Raman spectral features arising from differently oriented crystals.

### Quantum-Chemical Simulations

Simulations were performed to compare the calculated IR spectra with the experimentally obtained data. All calculations were carried out using *Gaussian 16* employing the B3LYP/6–311G­(d,p) basis set with full geometry optimization and frequency analysis (*opt freq* keyword).^39^ The molecular structures were initially drawn in *ChemSketch* and preoptimized in *Avogadro* ^40^ prior to the quantum-chemical calculations. The data were shown as absorption spectra 500 cm^–1^ to 2500 cm^–1^.

### FRAP Assay

The assay was performed using protocol of Dudonné et al.^30^ Briefly, FRAP reagent was mixed with 100 mL of 300 mM sodium acetate buffer with 10 mL of 10 mM TPTZ (2,4,6-tri­(2-pyridyl)-s-triazine) in 40 mM HCl and 10 mL 20 mM FeCl_3_. 100 μL of the samples was then mixed with 3 mL of FRAP reagent. After incubation for 30 min at 37 °C, absorbance of the mixture was measured at 593 nm. Ascorbic acid was used as positive control in the range of concentrations 0–200 μg/mL. The test was performed in duplicates.

### Cytotoxicity (MTT) Assay

Human breast cancer cell line (MDA-MB231), human colorectal carcinoma cell line (HCT116), and immortalized retinal pigment epithelial cells (hTERT-RPE-1) were obtained from the ATCC culture collection and used to assess cytotoxicity. Cells were maintained at 37 °C in a humidified atmosphere with 5% CO_2_ in a DMEM cultivation medium (Gibco Life Technologies) supplemented with 10% FBS, 1% antibiotics, and 1% l-glutamine 2 mM (all Gibco Life Technologies). The HCT116, MDA-MB231, and hTERT-RPE-1 cells were seeded into a 96-well white flat-bottom plate at a concentration of 5 × 10^3^ cells per well 1 day prior to the experiment and incubated at 37 °C in 5% CO_2_ atmosphere. After incubation, cells were treated at a total of 12 concentrations of **1**, **2** or **3** (using 2-fold dilution) in the concentration range 0–20 μM for 48 h. In the case of **2**, two additional concentration points were added to better delineate the dose response curve (3 μM and 1.75 μM). Subsequently, 10 μL MTT (4 mg/mL) solution was added to each well and incubated at 37 °C in 5% CO_2_ atmosphere for 4 h. At the end of the incubation period, the medium was removed and replaced with 200 μL DMSO in each well and allowed to stand for 20 min at the room temperature. Cell viability was quantified by measuring absorbance at 590 nm with a reference wavelength of 640 nm using a TECAN SUNRISE microplate reader (Schoeller instruments). Medium containing 1% DMSO served as the negative control, and nostatin A was used as the positive control.^41^ Assays were performed in triplicates.

## Supplementary Material



## Data Availability

The NMR data for synurins A–C (**1–3**) have been deposited in the Natural Products Magnetic Resonance Database (NP-MRD; www.np-mrd.org) and can be found at NP0353915 (**1**, synurin A), NP0353916 (**2**, synurin B), and NP0353917 (**3**, synurin C). FTICR data are available at DOI: 10.5281/zenodo.19911757. HPLC-HRMS, FTIR, and Raman spectroscopy data sets are available in ZENODO under DOI: 10.5281/zenodo.20020731. The remaining data that support the findings of this study are available from the corresponding author upon reasonable request.

## Notes

HRMS/MS spectra for all compounds; NMR spectra (^1^H, HSQC, HMBC, ^13^C APT for all compounds; COSY for compound **1**; NOESY for compounds **2** and **3**); FT-IR MS spectra for all compounds; FT-ICR MS spectrum for compound **2**; brightfield and polarization images of all compounds formed from MeOH solutions; dose-dependent viability of cell lines treated with all compounds; HPLC traces of all compounds; selected natural NQ products (including references) structurally related to all compounds (PDF)
