# Supplementary material for: Cytotoxic Synurins A–C: Chlorinated Naphthoquinone Pigments from the Freshwater Alga Synura sphagnicola
Source: J Nat Prod. 2026 May 26;89(6):1932–41. doi: 10.1021/acs.jnatprod.6c00417 (PMC13316993; doi:10.1021/acs.jnatprod.6c00417)
Supplement: Supplementary file 1 [file np6c00417_si_001.pdf]

Supporting Information

Cytotoxic Synurins A–C: Chlorinated Naphthoquinone Pigments from the Freshwater Alga *Synura sphagnicola*

Magda Škaloudová[1], Jan Blahut[2], Jan Hájek[3], Alan Kádek[4], Peter Mojzeš[5], Jana Pilátová[5,6] , Dominika Tučková[3,7], Petra Divoká[3,7], Antonín Střížek[3], Martin Lukeš[3], Eva Kotabová[3], Petra Bittnerová[7], Kumar Saurav[3], Pavel Hrouzek[3] and Petra Urajová[3*]

[1]Faculty of Science, Charles University, 128 43 Prague, Czech Republic

[2]Institute of Organic Chemistry and Biochemistry, Czech Academy of Sciences, 160 00 Prague, Czech Republic

[3]Centre Algatech, Institute of Microbiology of the Czech Academy of Sciences, 379 01 Třeboň, Czech Republic

[4]BIOCEV, Institute of Microbiology of the Czech Academy of Sciences, 252 50 Vestec, Czech Republic

[5]Institute of Physics, Faculty of Mathematics and Physics, Charles University, 128 43 Prague, Czech Republic

[6] Institute of Parasitology, Biology Centre, Czech Academy of Science, 370 05 České Budějovice, Czech Republic

[7]Faculty of Science, University of South Bohemia, 370 05 České Budějovice, Czech Republic

*** Corresponding author**

Dr. Petra Urajová

Institute of Microbiology, CAS
Centre Algatech
Novohradská 237 - Opatovický mlýn
37901 Třeboň, Czech Republic

e-mail: urajova@alga.cz

List of supporting information

FIG. S1. ESI-HRMS SPECTRA OF COMPOUNDS **1–3.** 3
FIG. S2. HRMS/MS SPECTRA OF COMPOUNDS **1–3**. 4
FIG. S3. ^{1}H SPECTRUM ($CDCL_3$, 500 MHZ, 25˚C) OF COMPOUND **1**. 5
FIG. S4. COSY SPECTRUM ($CDCL_3$, 500 MHZ, 25˚C) OF COMPOUND **1**. 6
FIG. S5. ^{1}H-^{13}C HSQC SPECTRUM ($CDCL_3$, 600 MHZ/126 MHZ, 25˚C) OF COMPOUND **1**. 7
FIG. S6. ^{1}H-^{13}C HMBC SPECTRUM ($CDCL_3$, 600 MHZ/126 MHZ, 25˚C) OF COMPOUND **1**. 8
FIG. S7. ^{13}C APT SPECTRUM ($CDCL_3$, 126 MHZ, 25˚C) OF COMPOUND **1**. 9
FIG. S8. FOURIER-TRANSFORMATION INFRARED SPECTRA OF COMPOUNDS **1–3**. 10
FIG. S9. ULTRA-HIGH RESOLUTION FT-ICR MS SPECTRUM OF COMPOUND **2** INDICATING PRESENCE OF CHLORINE IN THE MOLECULE THROUGH ISOTOPIC FINE STRUCTURE PEAK FITTING. 11
FIG. S10. ^{1}H SPECTRUM ($CDCL_3$, 500 MHZ, 25˚C) OF COMPOUND **2**. 12
FIG. S11. ^{13}C APT SPECTRUM ($CDCL_3$, 126 MHZ, 25˚C) OF COMPOUND **2**. 13
FIG. S12. ^{1}H-^{13}C HSQC SPECTRUM ($CDCL_3$, 600 MHZ/126 MHZ, 25˚C) OF COMPOUND **2**. 14
FIG. S13. ^{1}H-^{13}C HMBC SPECTRUM (CDCL3, 600 MHZ/126 MHZ, 25˚C) OF COMPOUND **2**. 15
FIG. S14. ^{1}H SPECTRUM OF **3** ($CDCL_3$, 500MHZ, 25˚C). 16
FIG. S15. ^{13}C APT SPECTRUM OF **3** ($CDCL_3$, 126MHZ, 25˚C). 17
FIG. S16. ^{1}H-^{13}C HSQC SPECTRUM OF **3** ($CDCL_3$, 600MHZ/126MHZ, 25˚C). 18
FIG. S17. ^{1}H-^{13}C HMBC SPECTRUM OF **3** ($CDCL_3$, 600MHZ/126MHZ, 25˚C). 19
FIG. S18. COMPARISON OF ^{1}H NMR SPECTRA ($CDCL_3$, 500 MHZ, 25˚C) OF **1–3.** COMPOUND **2** WITH INSETS OF STRUCTURAL MOTIVES CORRESPONDING TO GIVEN REGIONS IN THE SPECTRUM. 20
FIG. S19. DETAIL OF 1D GRADIENT SELECTIVE NOESY (RED) OVERLAYED WITH 1H SPECTRA FOR COMPOUND 2 AND 3 USING 300 MS MIXING TIME AND SELECTIVE PULSE ON-RESONANCE WITH CH_3 GROUP (H-14 AND H-15 FOR 2 AND 3, RESPECTIVELY) (500 MHZ, $CDCL_3$). 21
FIG. S20. REPRESENTATIVE BRIGHTFIELD AND POLARIZATION IMAGES OF **1** FORMED FROM MEOH SOLUTIONS. 22
FIG. S21. REPRESENTATIVE BRIGHTFIELD AND POLARIZATION IMAGES OF **2** FORMED FROM MEOH SOLUTIONS. 23
FIG. S22. REPRESENTATIVE BRIGHTFIELD AND POLARIZATION IMAGES OF **3** FORMED FROM MEOH SOLUTIONS. 24
FIG. S23. DOSE-DEPENDENT VIABILITY OF A) HCT116, B) MDA-MB-231, AND C) HTERT-RPE-1 CELL LINES TREATED BY **1**–**3**. 25
FIG. S24. HPLC-DAD CHROMATOGRAM OF **1**–**3**. 26
TABLE S1. LIST OF DETECTED CORRELATION FOR COMPOUND **1** 27
TABLE S2. SELECTED NATURAL NQ PRODUCTS STRUCTURALLY RELATED TO **1–3** 28
SI REFERENCES 30

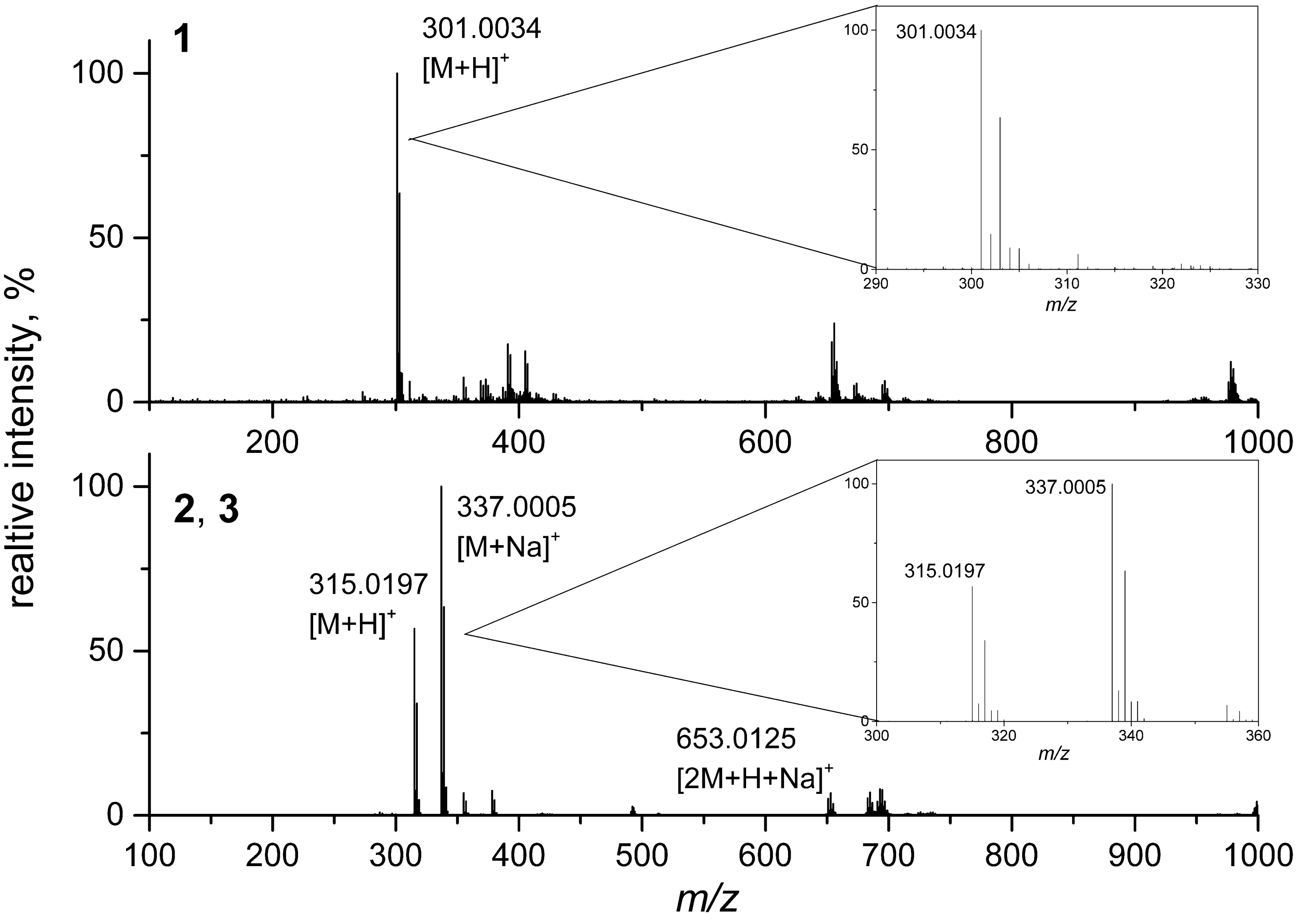

Fig. S1. ESI-HRMS spectra of compounds **1–3.**

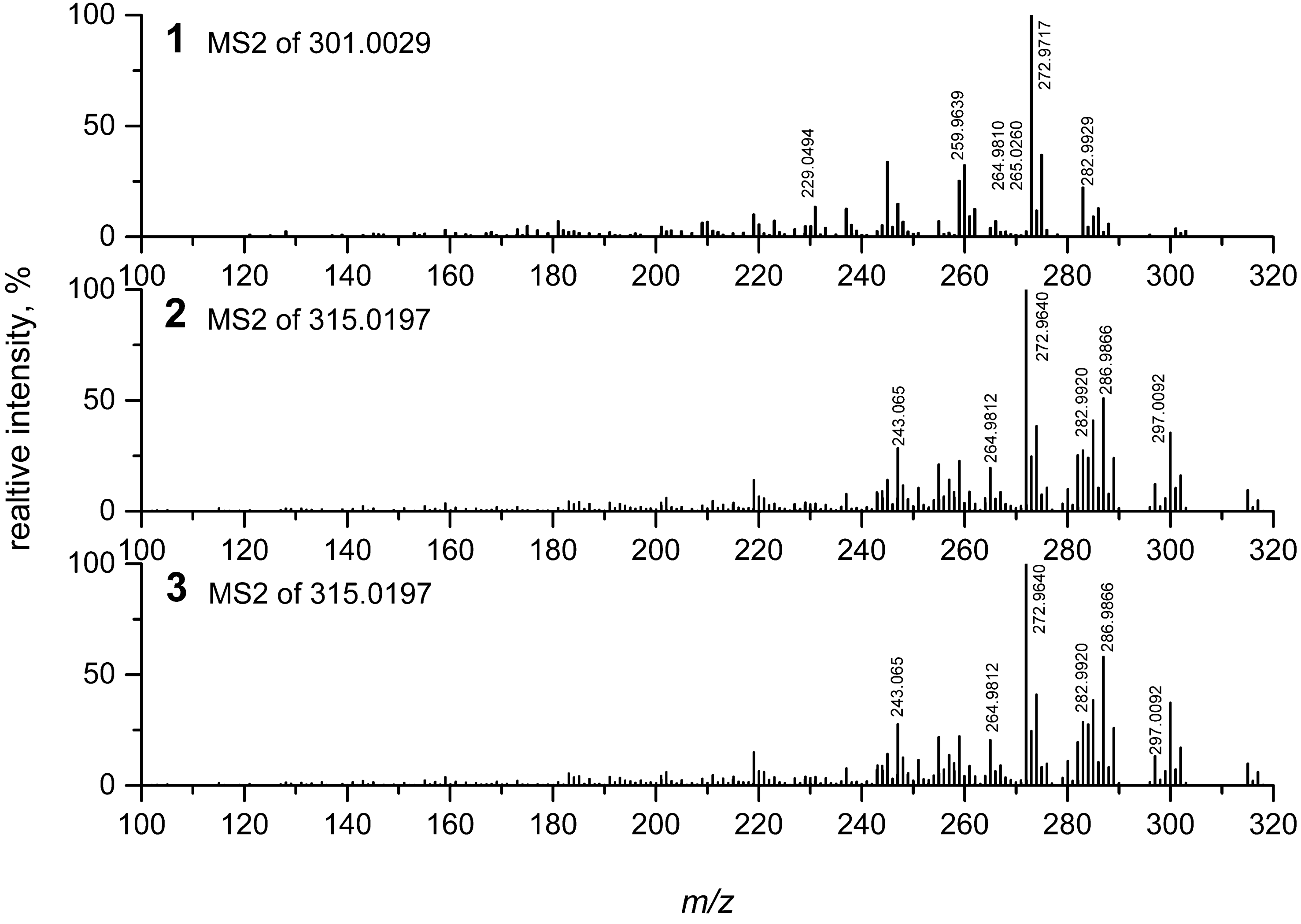

Fig. S2. HRMS/MS spectra of compounds **1–3** (a-c).

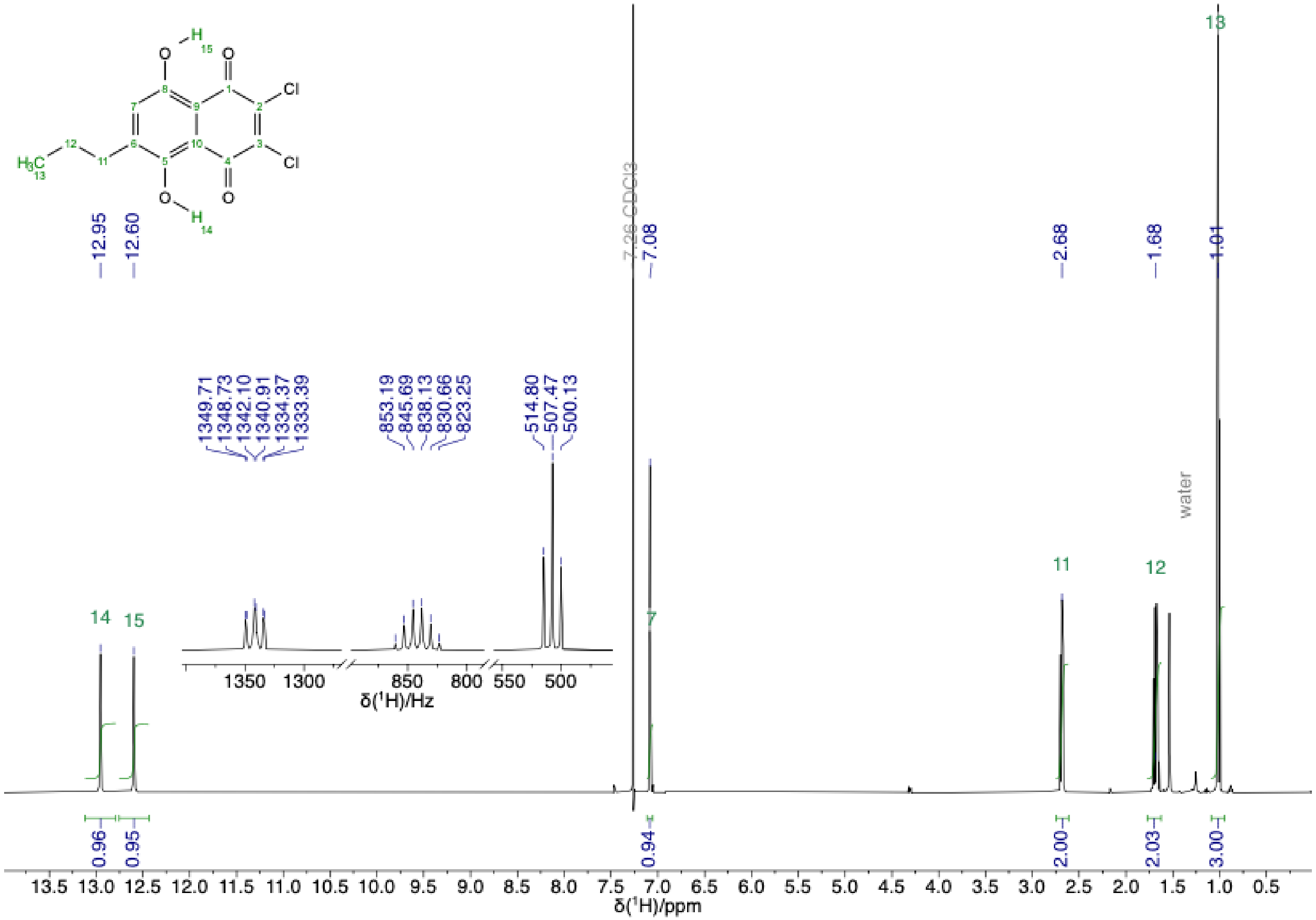

Fig. S3. ^{1}H spectrum ($CDCl_3$, 500 MHz, 25˚C) of compound **1**.

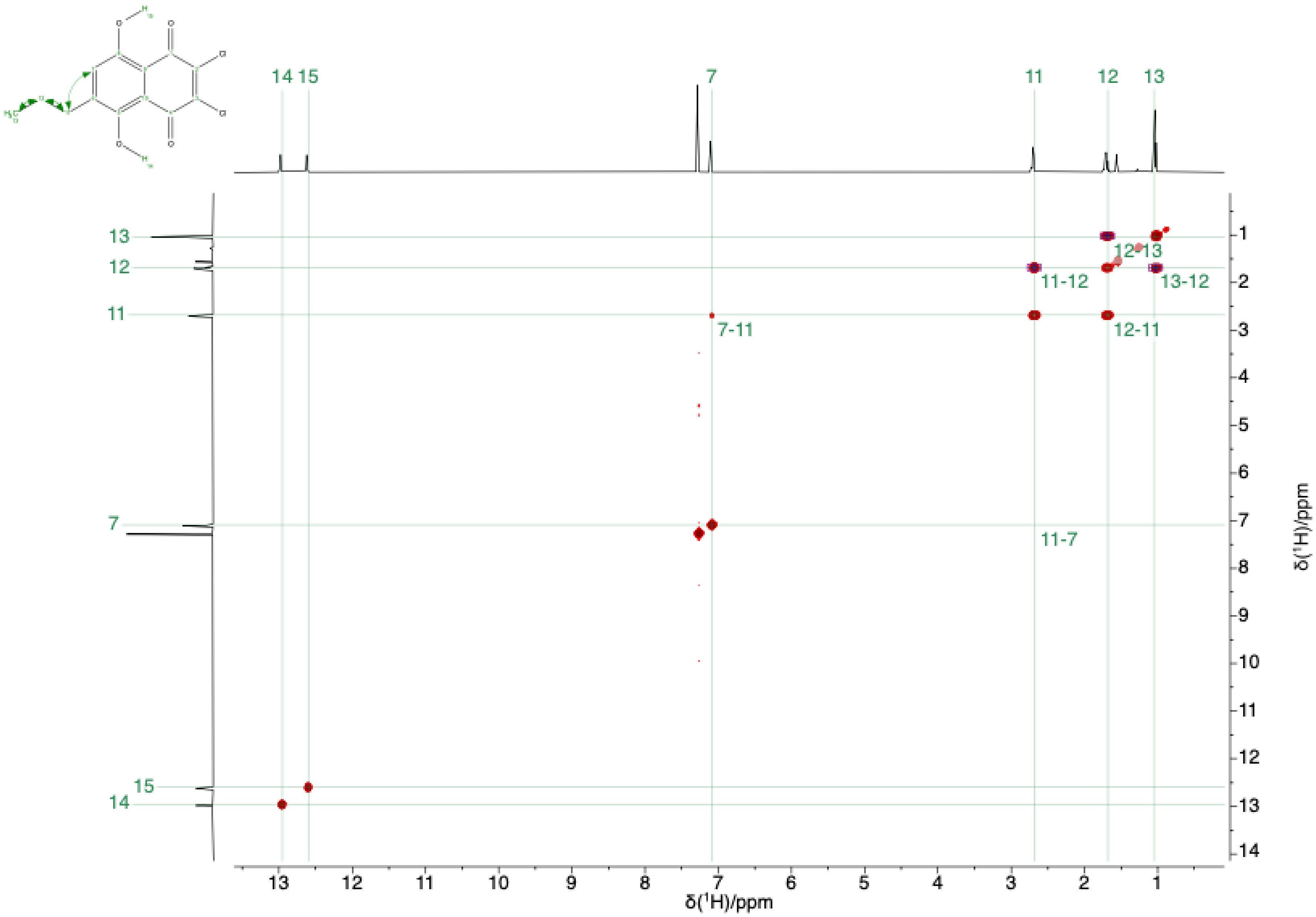

Fig. S4. COSY spectrum ($CDCl_3$, 500 MHz, 25˚C) of compound **1**.

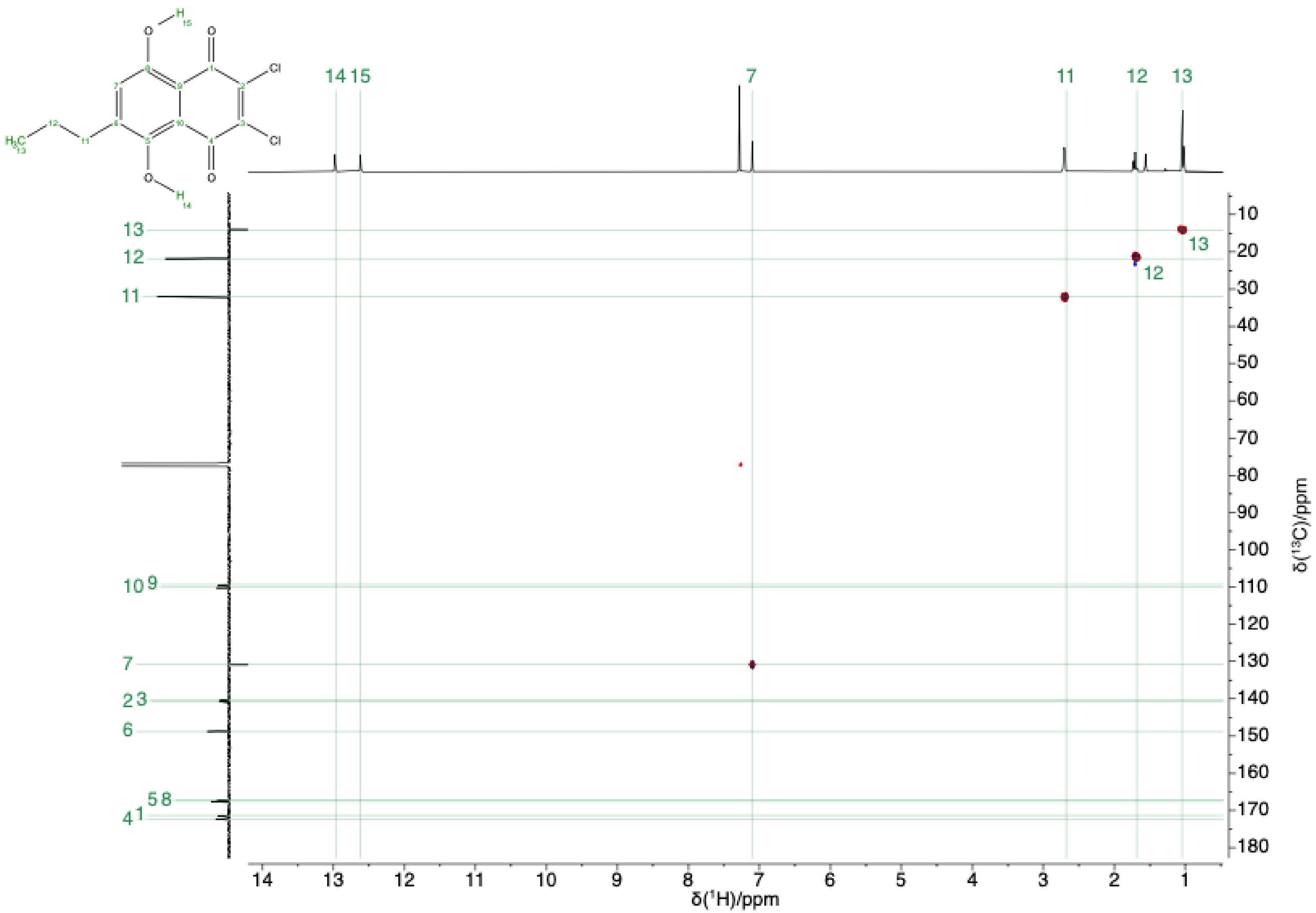

Fig. S5. ^{1}H-^{13}C HSQC spectrum ($CDCl_3$, 600 MHz/126 MHz, 25˚C) of compound **1**.

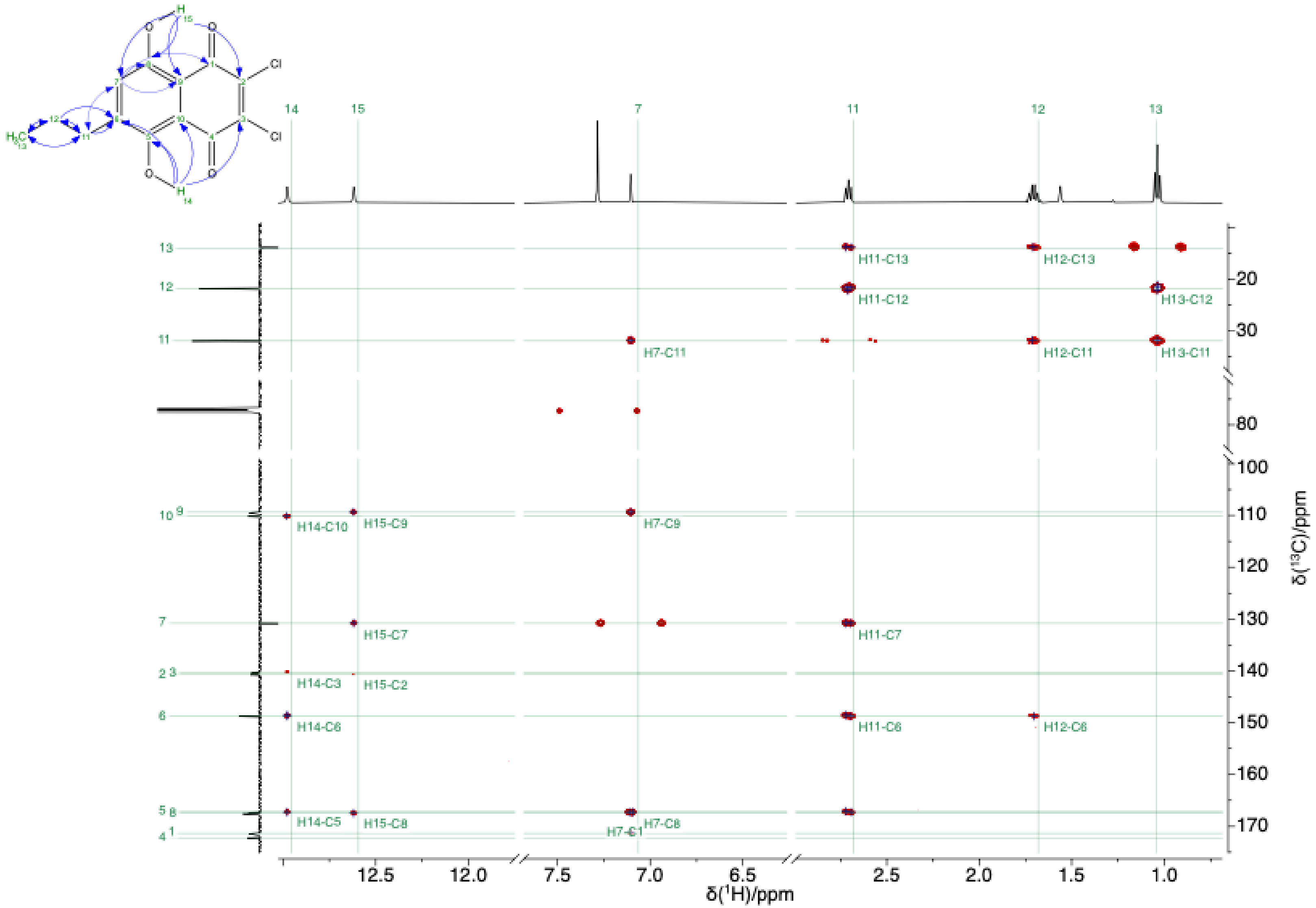

Fig. S6. ^{1}H-^{13}C HMBC spectrum (CDCl₃, 600 MHz/126 MHz, 25˚C) of compound **1**.

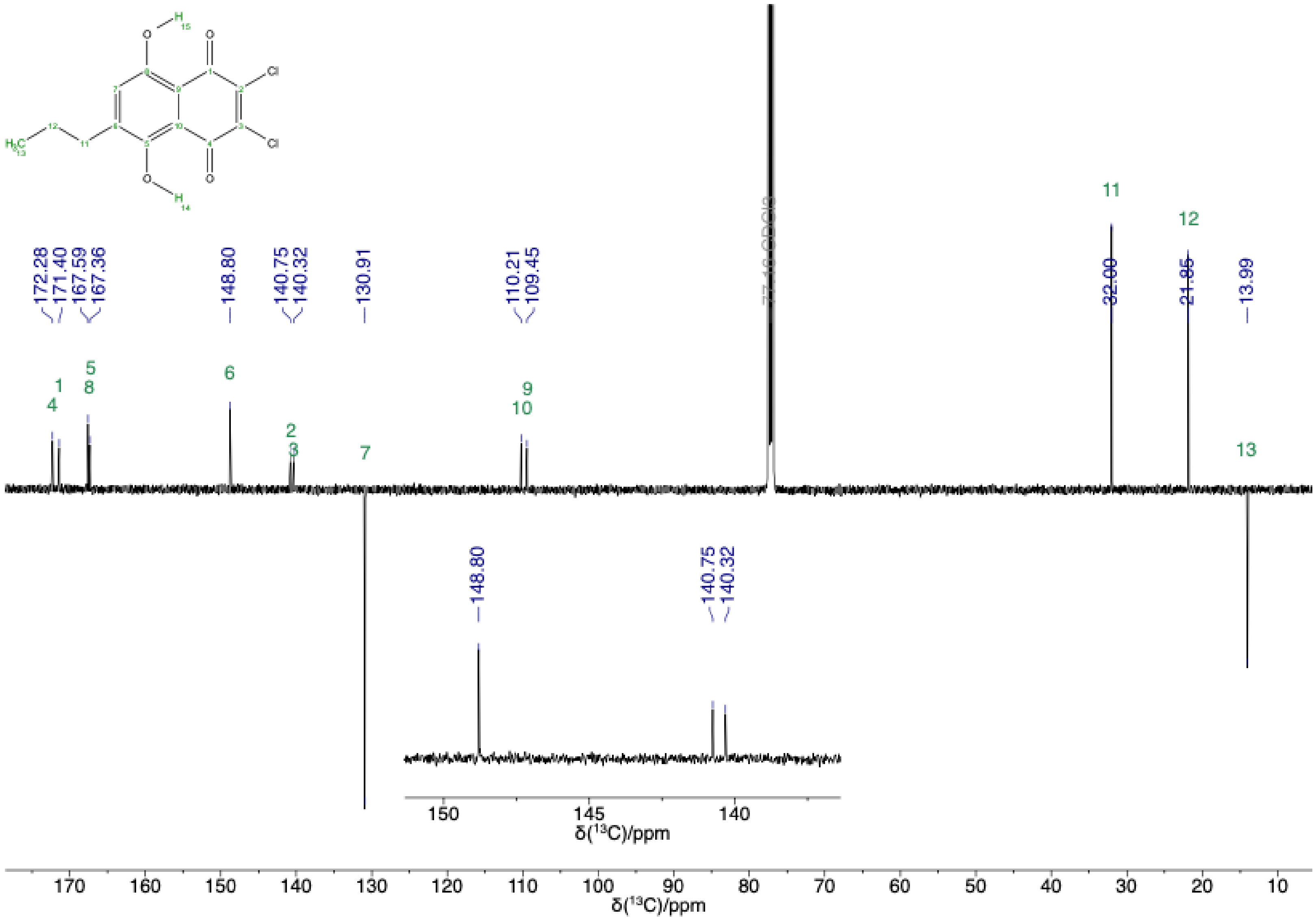

Fig. S7. ^{13}C APT spectrum ($CDCl_3$, 126 MHz, 25˚C) of compound **1**.

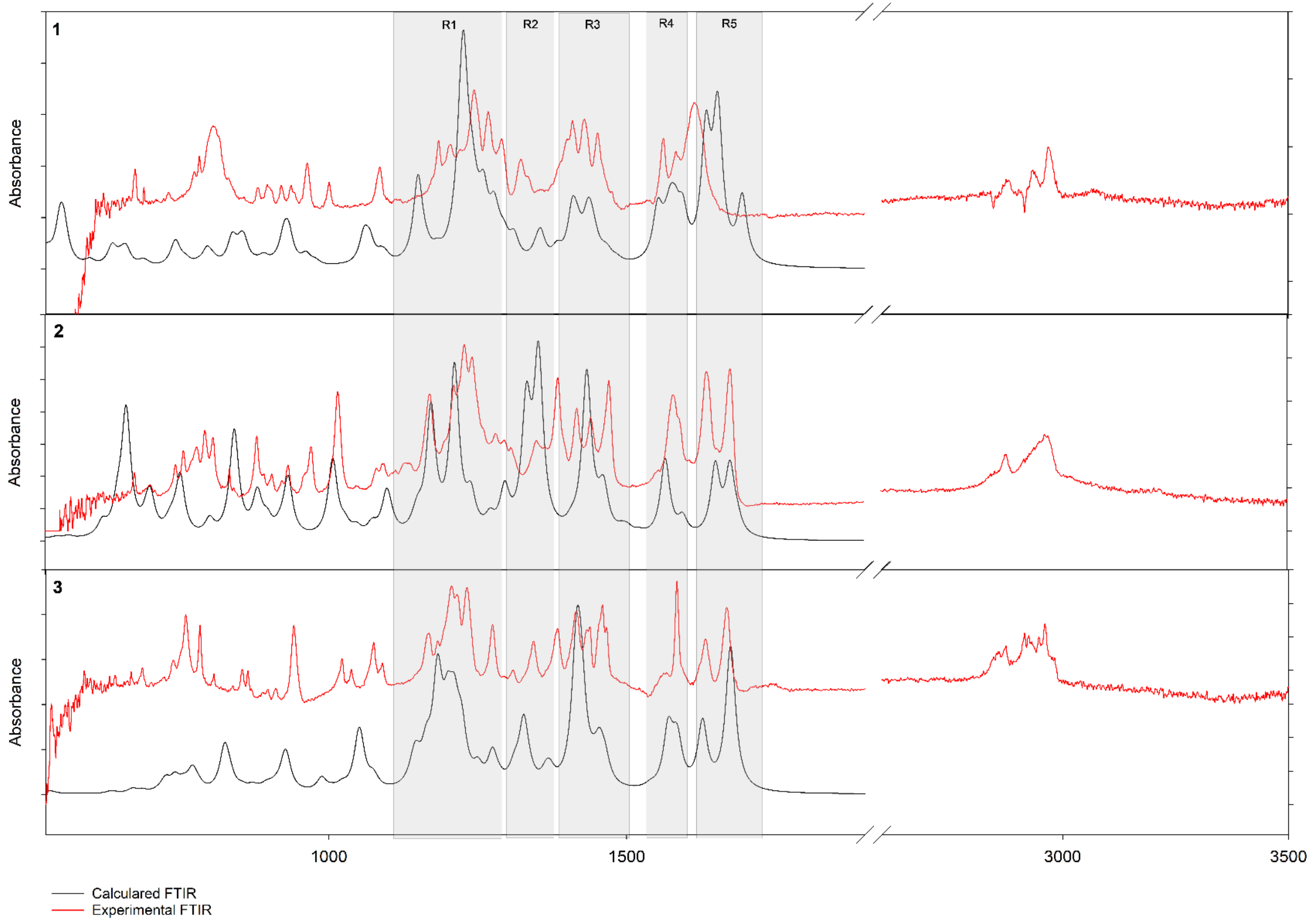

Fig. S8. Fourier-transformation infrared spectra of compounds **1–3**. Regions of particular vibrations bands are highlighted: R1 (1150–1220 cm^{-1}), R2 (1250–1280 cm^{-1}), R3 (1300–1500 cm^{-1}), R4 (1530–1550 cm^{-1}) and R5 (1625–1700 cm^{-1}).

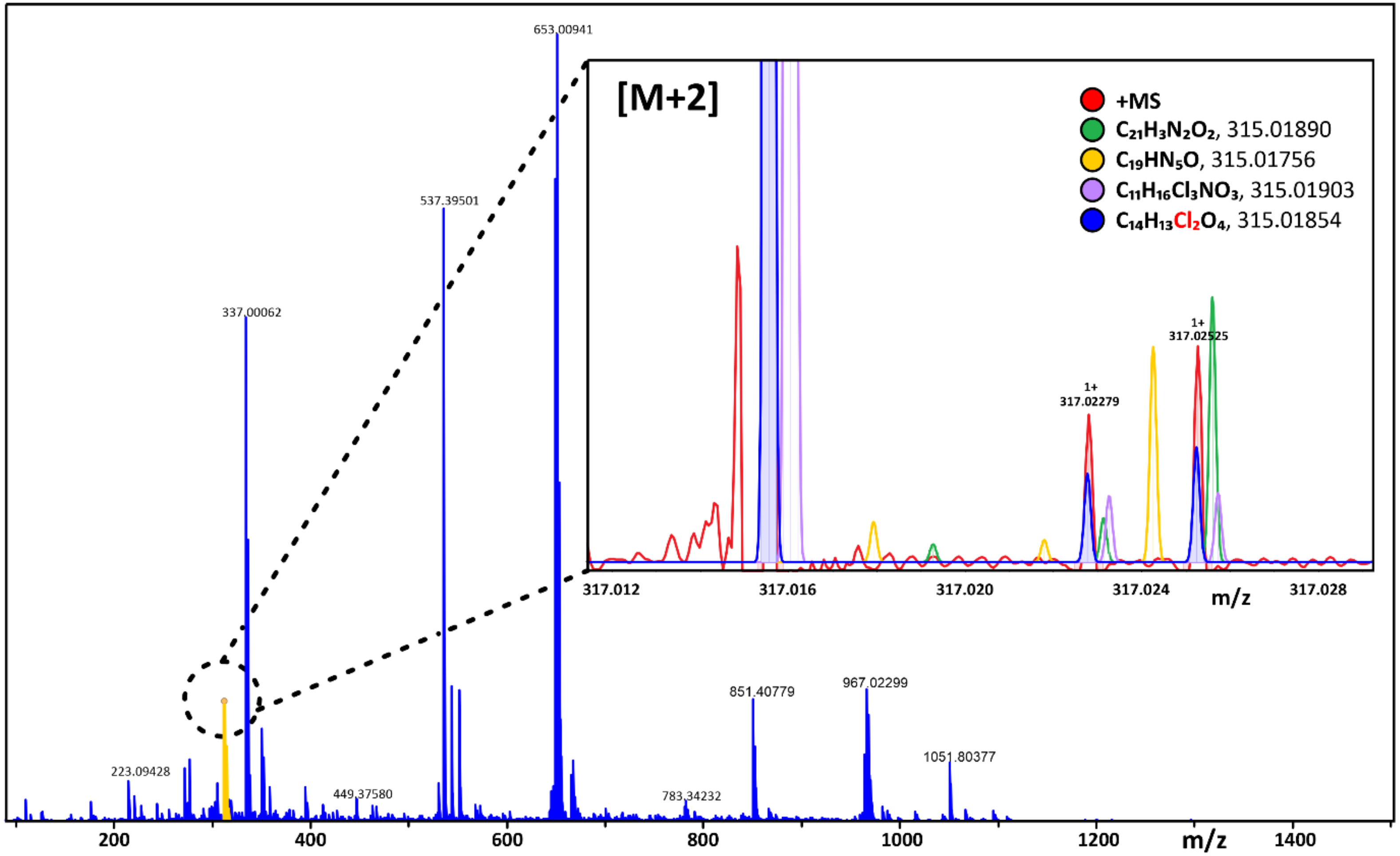

Fig. S9. Ultra-high resolution FT-ICR MS spectrum of compound **2** indicating presence of chlorine in the molecule through isotopic fine structure peak fitting. Fit of several closest candidate molecular formulas shown for the third isotopic peak [Mmono+2] in the inset.

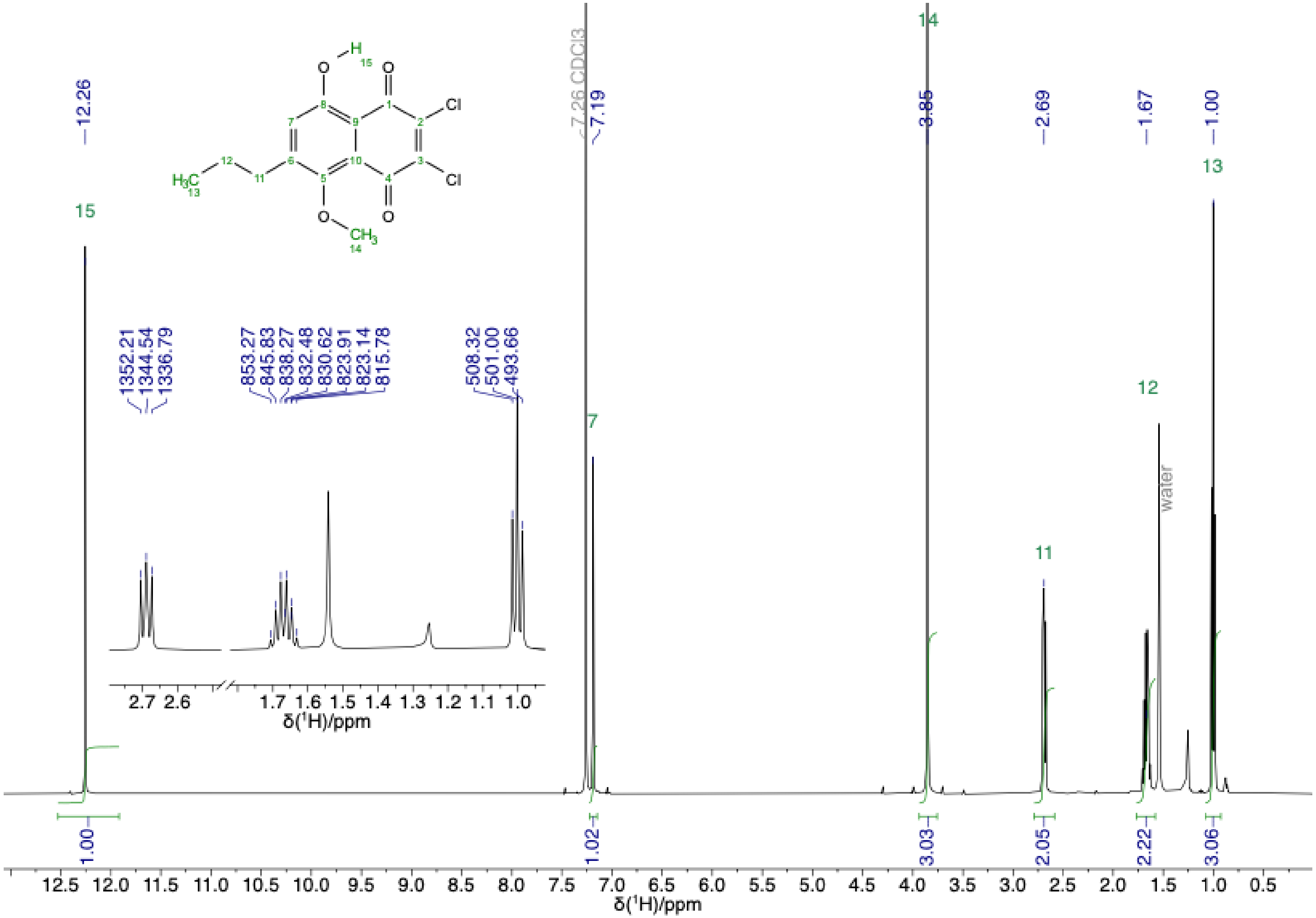

Fig. S10. ^{1}H spectrum ($CDCl_3$, 500 MHz, 25˚C) of compound **2**.

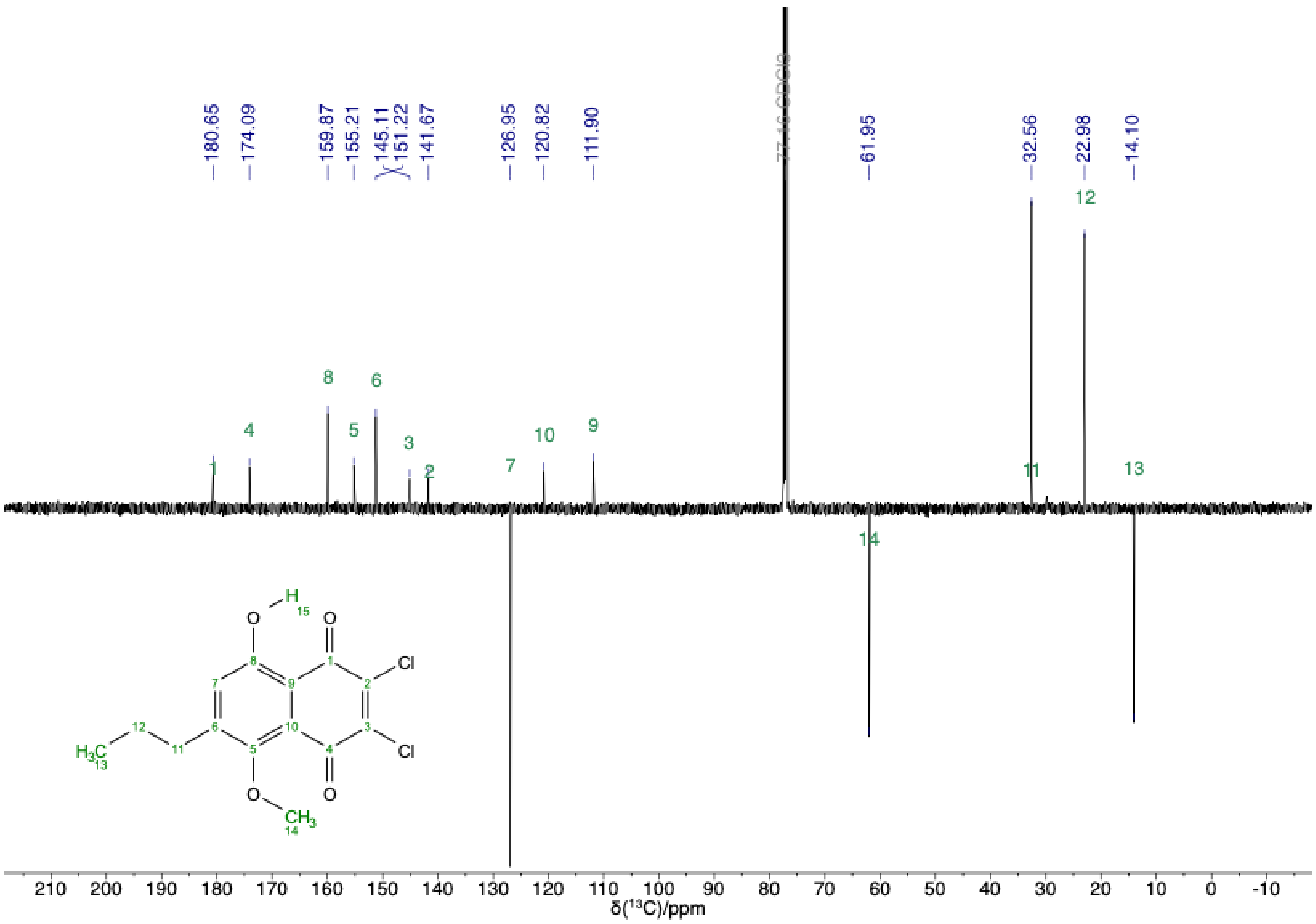

Fig. S11. ^{13}C APT spectrum (CDCl$_3$, 126 MHz, 25˚C) of compound **2**.

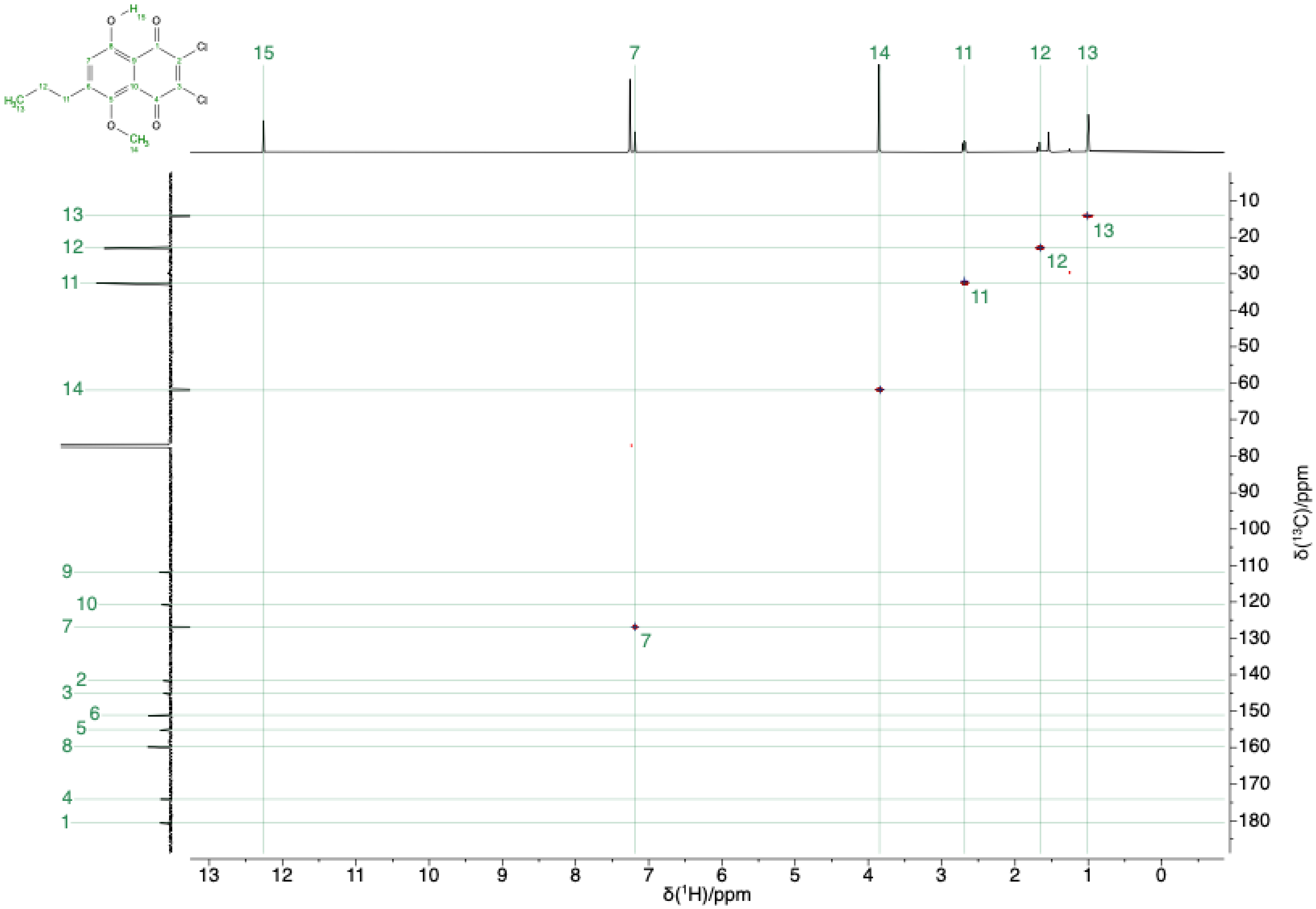

Fig. S12. ^{1}H-^{13}C HSQC spectrum ($CDCl_3$, 600 MHz/126 MHz, 25˚C) of compound **2**.

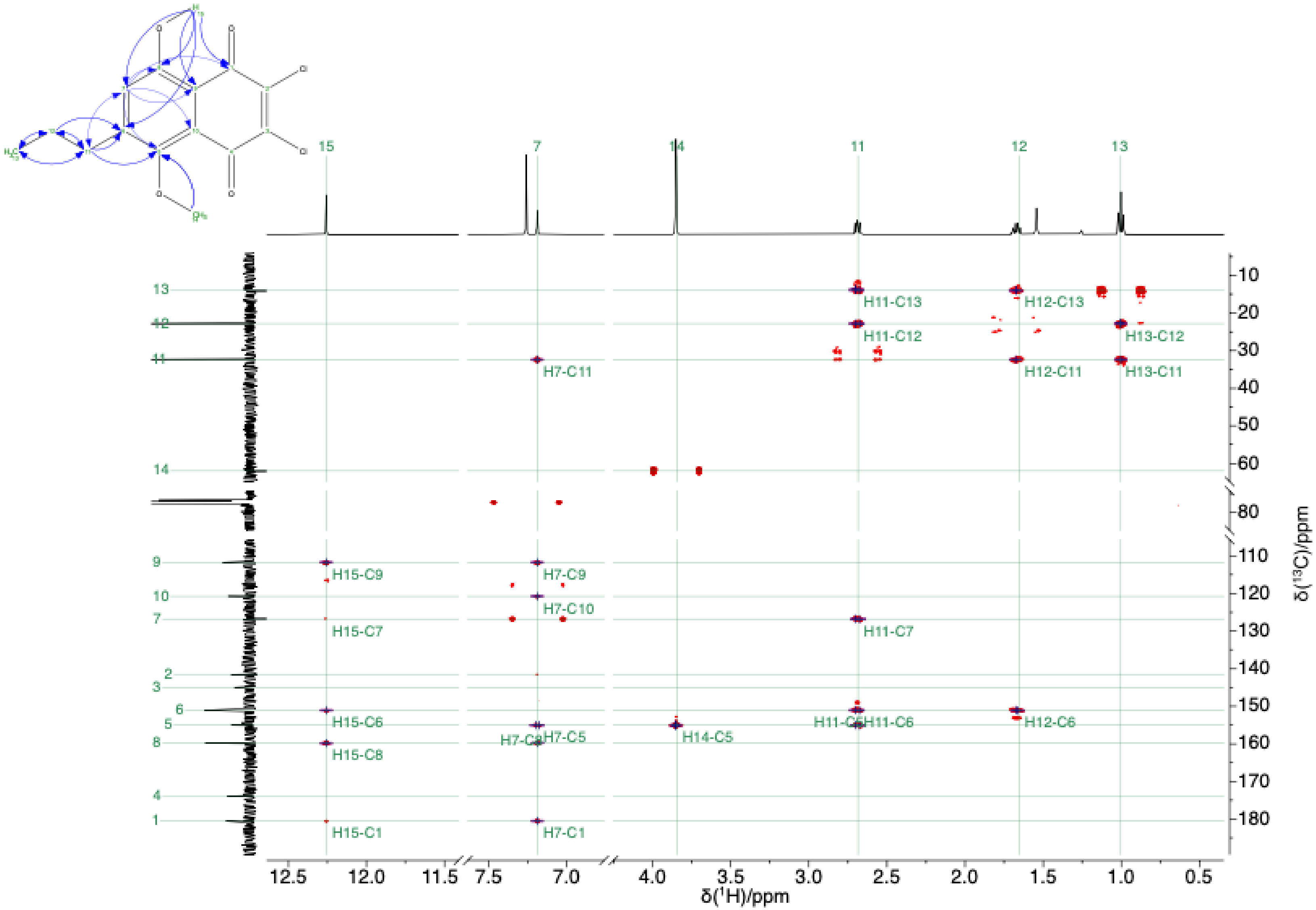

Fig. S13. ^{1}H-^{13}C HMBC spectrum (CDCl3, 600 MHz/126 MHz, 25˚C) of compound **2**.

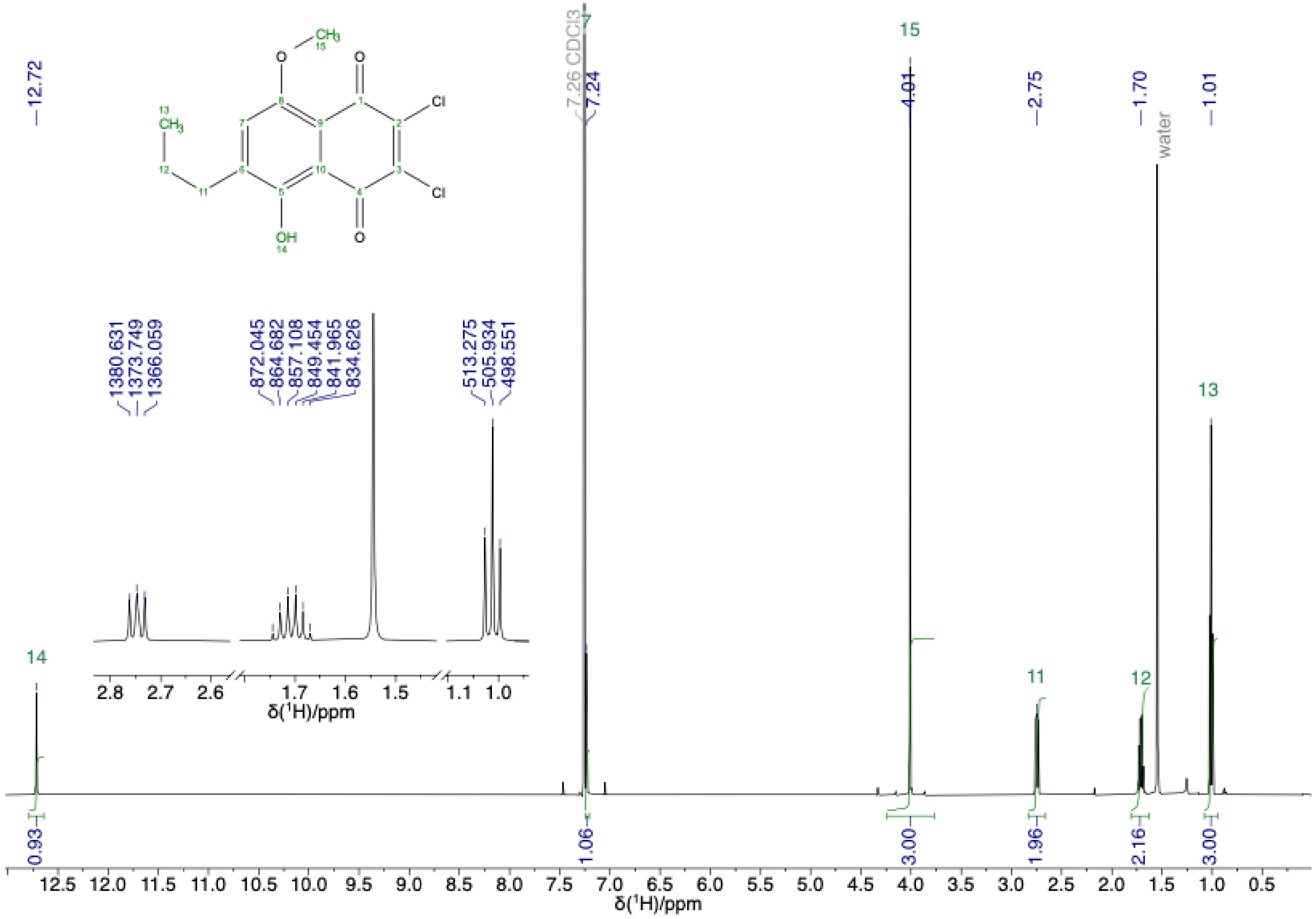

Fig. S14. ^{1}H spectrum of **3** ($CDCl_3$, 500MHz, 25°C).

.

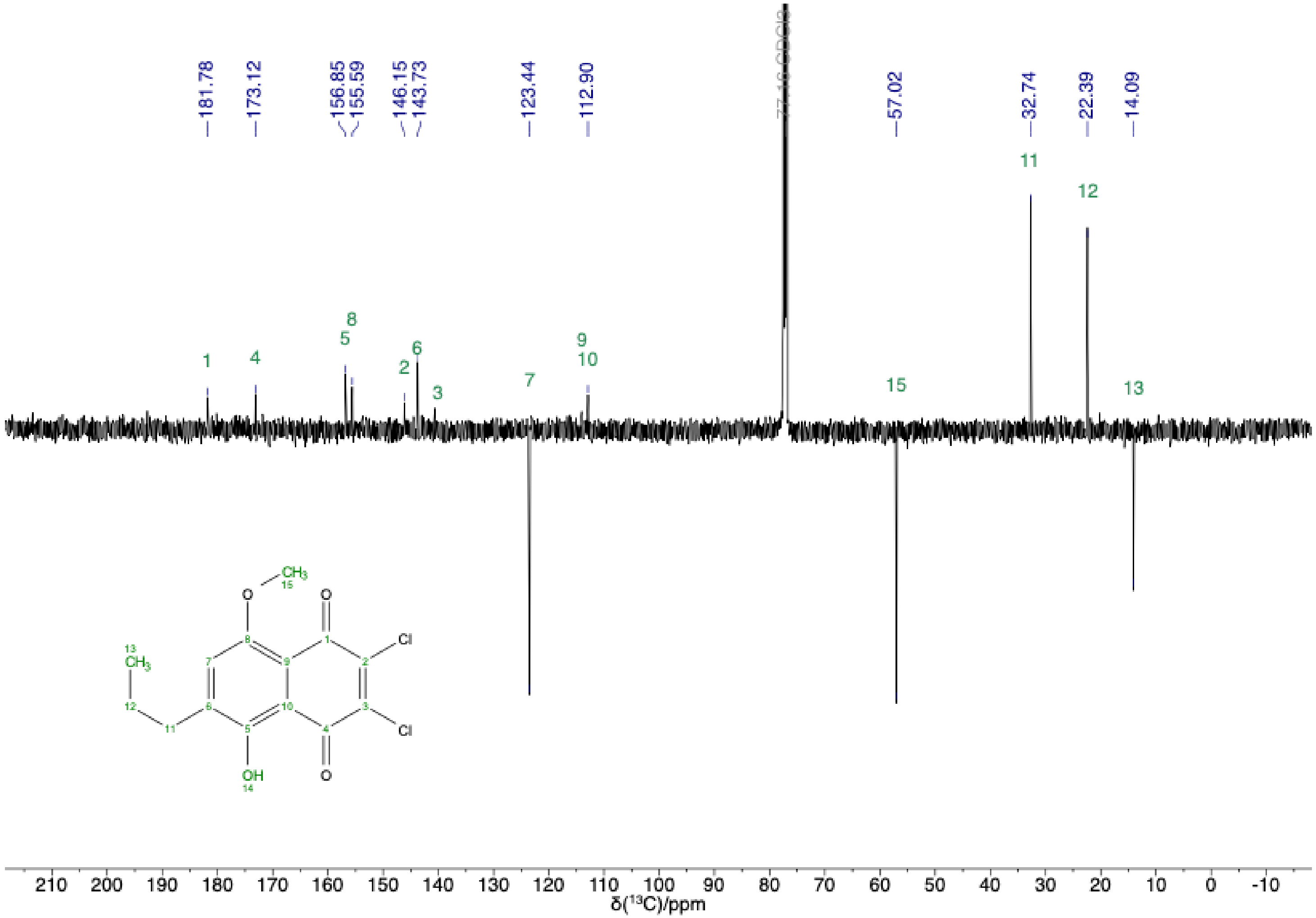

Fig. S15. ^{13}C APT spectrum of **3** ($CDCl_3$, 126MHz, 25˚C).

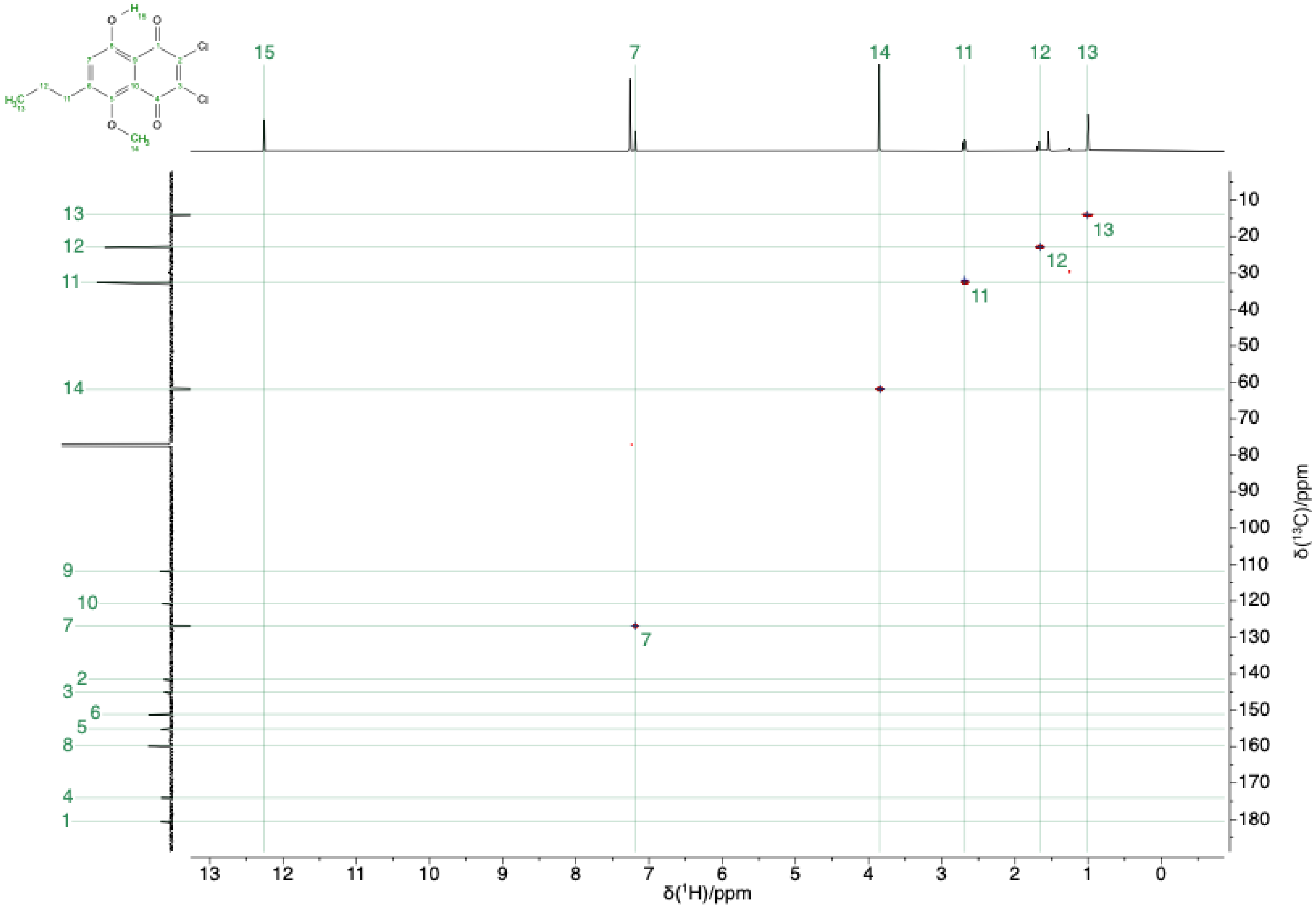

Fig. S16. ^{1}H-^{13}C HSQC spectrum of **3** ($CDCl_3$, 600MHz/126MHz, 25˚C).

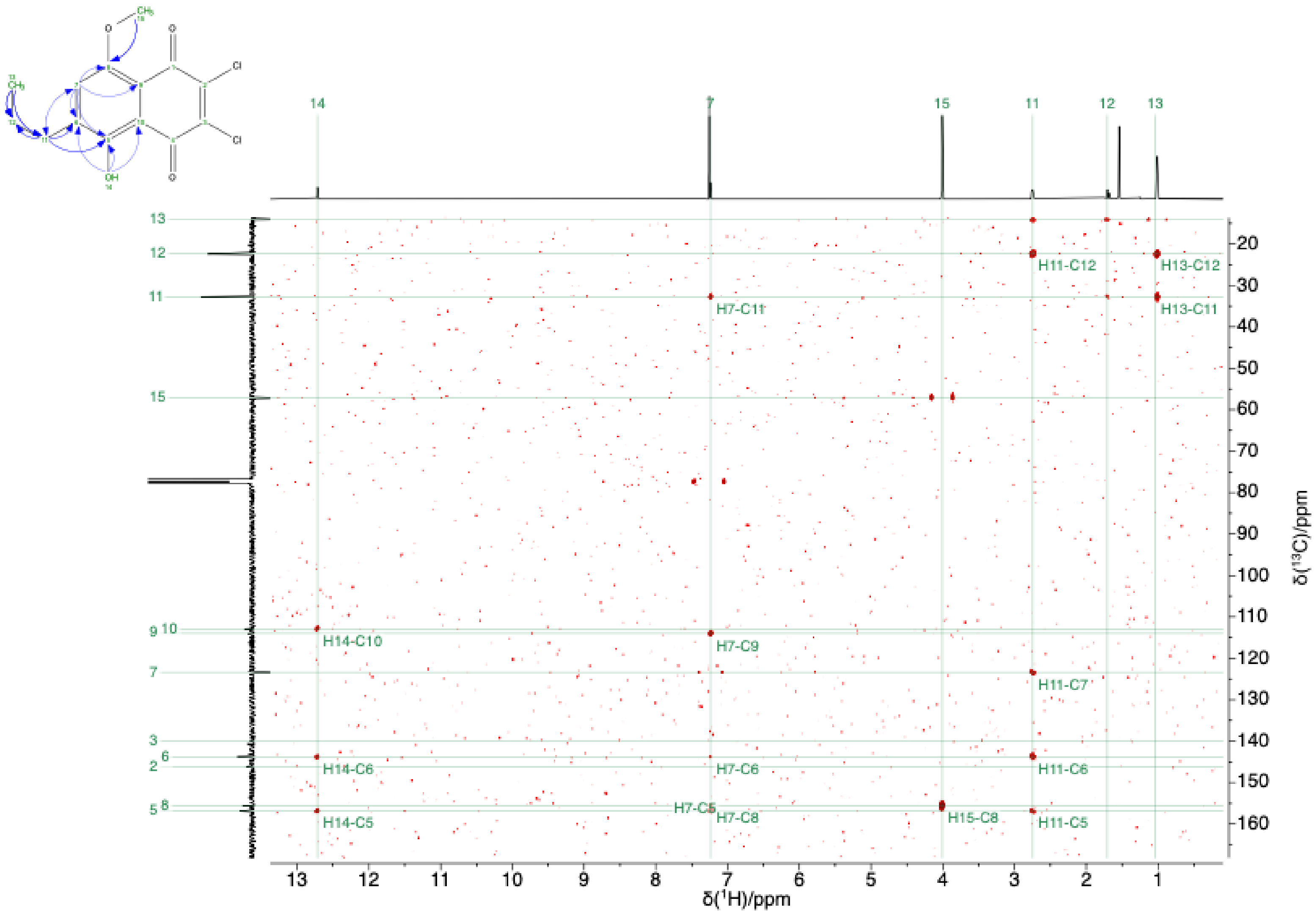

Fig. S17. ^{1}H-^{13}C HMBC spectrum of **3** ($CDCl_3$, 600MHz/126MHz, 25°C).

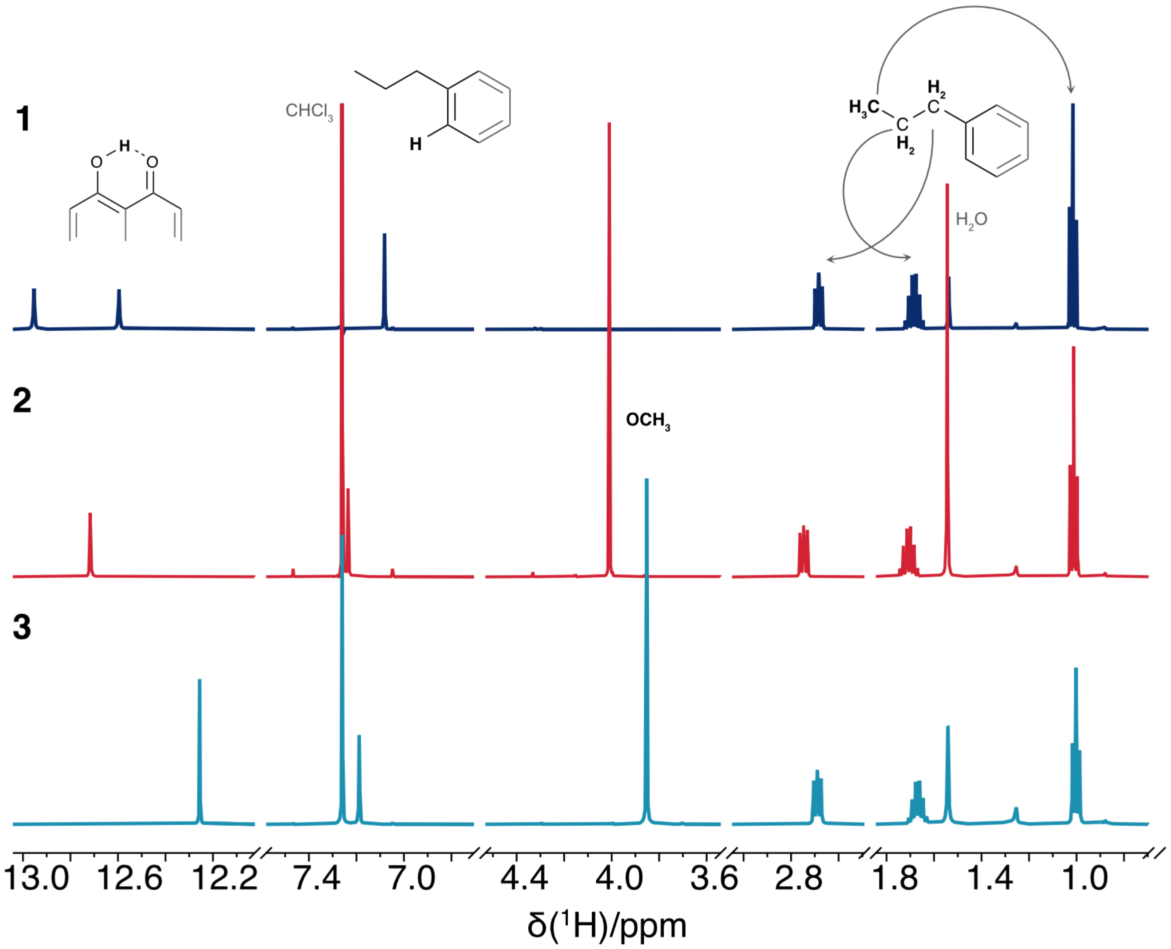

Fig. S18. Comparison of ^{1}H NMR spectra ($CDCl_3$, 500 MHz, 25˚C) of **1–3.** Compound **2** with insets of structural motives corresponding to given regions in the spectrum.

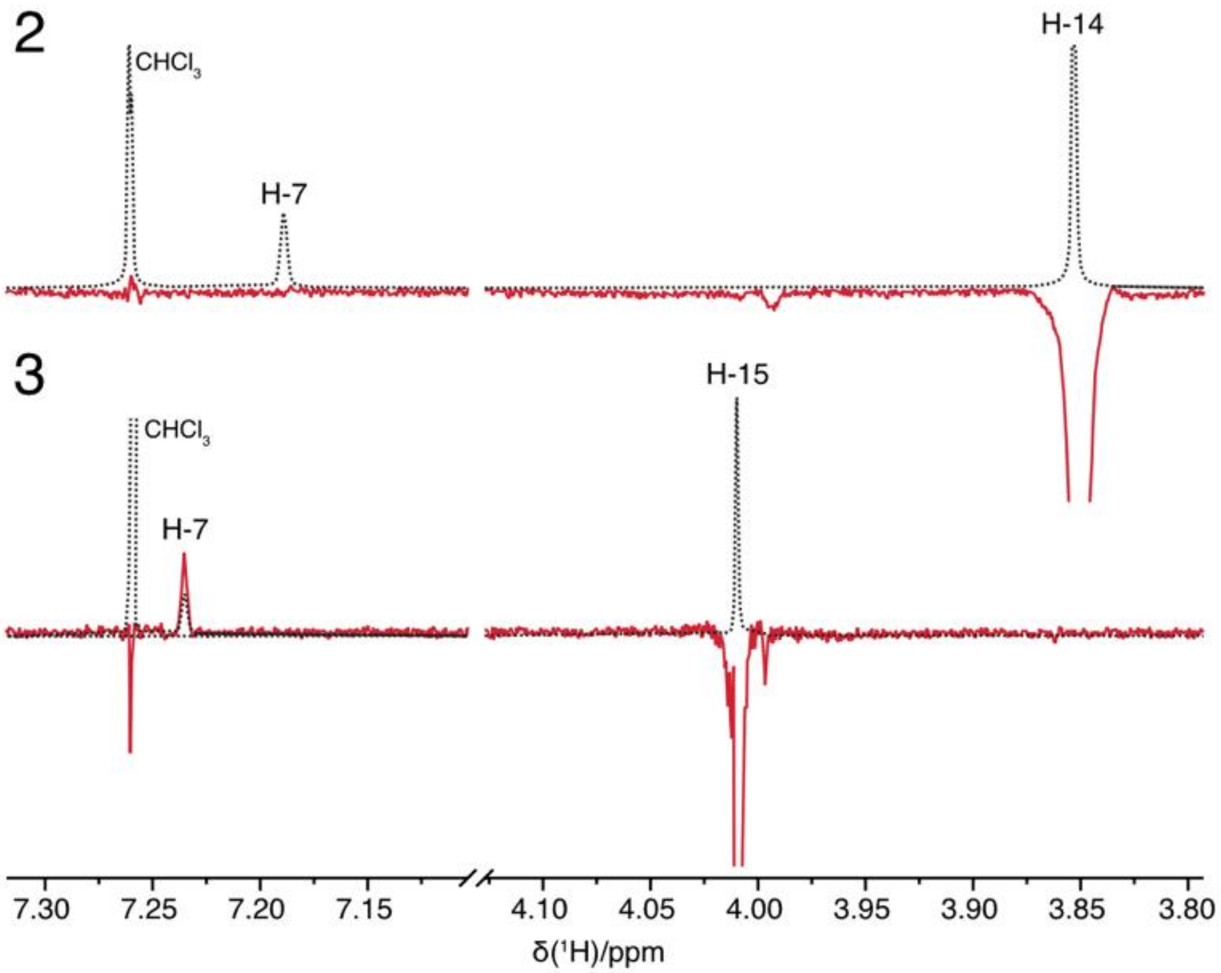

Fig. S19. Detail of 1D gradient selective NOESY (red) overlayed with 1H spectra for compound **2** and **3** using 300 ms mixing time and selective pulse on-resonance with CH_3 group (H-14 and H-15 for 2 and 3, respectively) (500 MHz, $CDCl_3$).

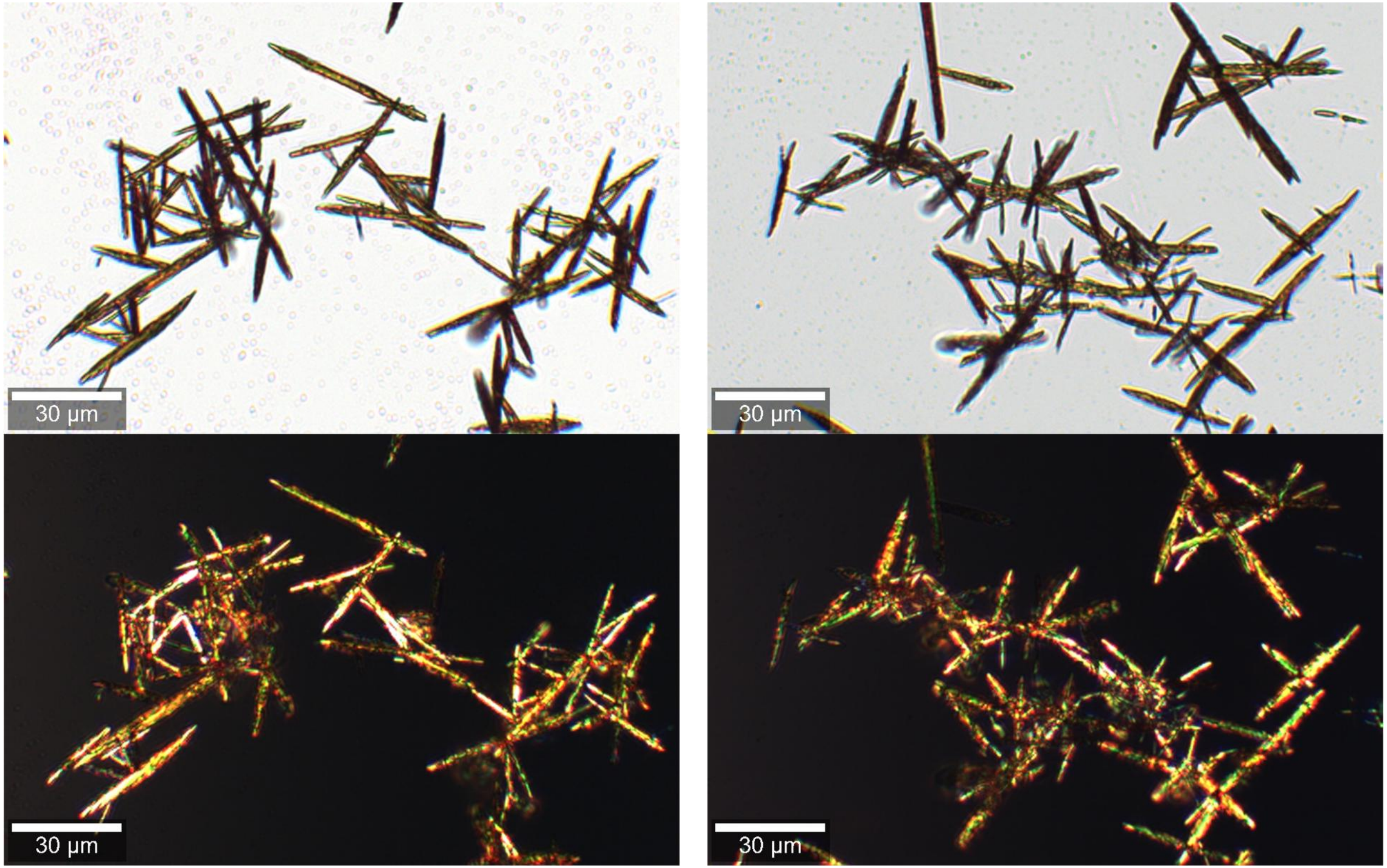

Fig. S20. Representative brightfield and polarization images of **1** formed from MeOH solutions.

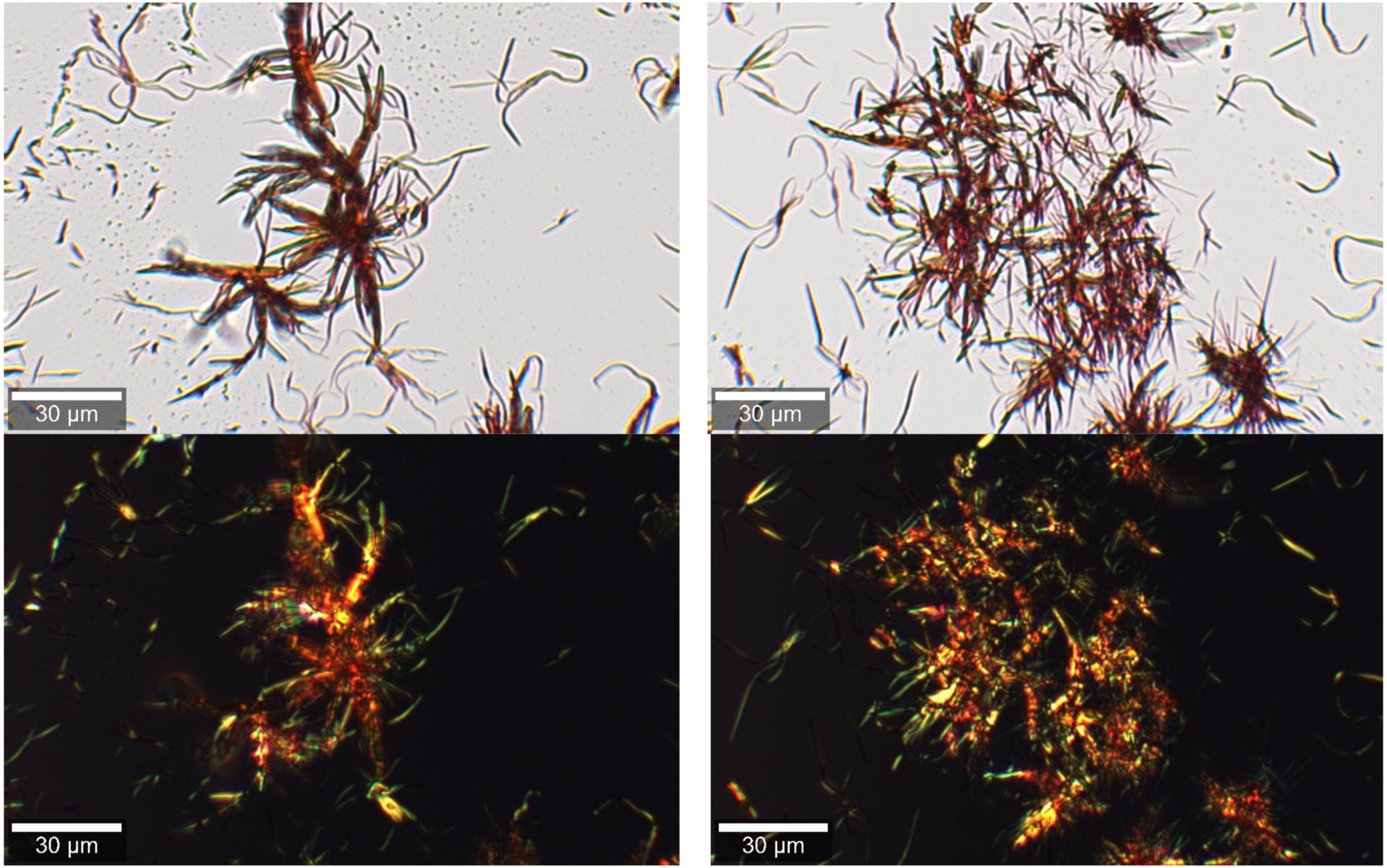

Fig. S21. Representative brightfield and polarization images of **2** formed from MeOH solutions.

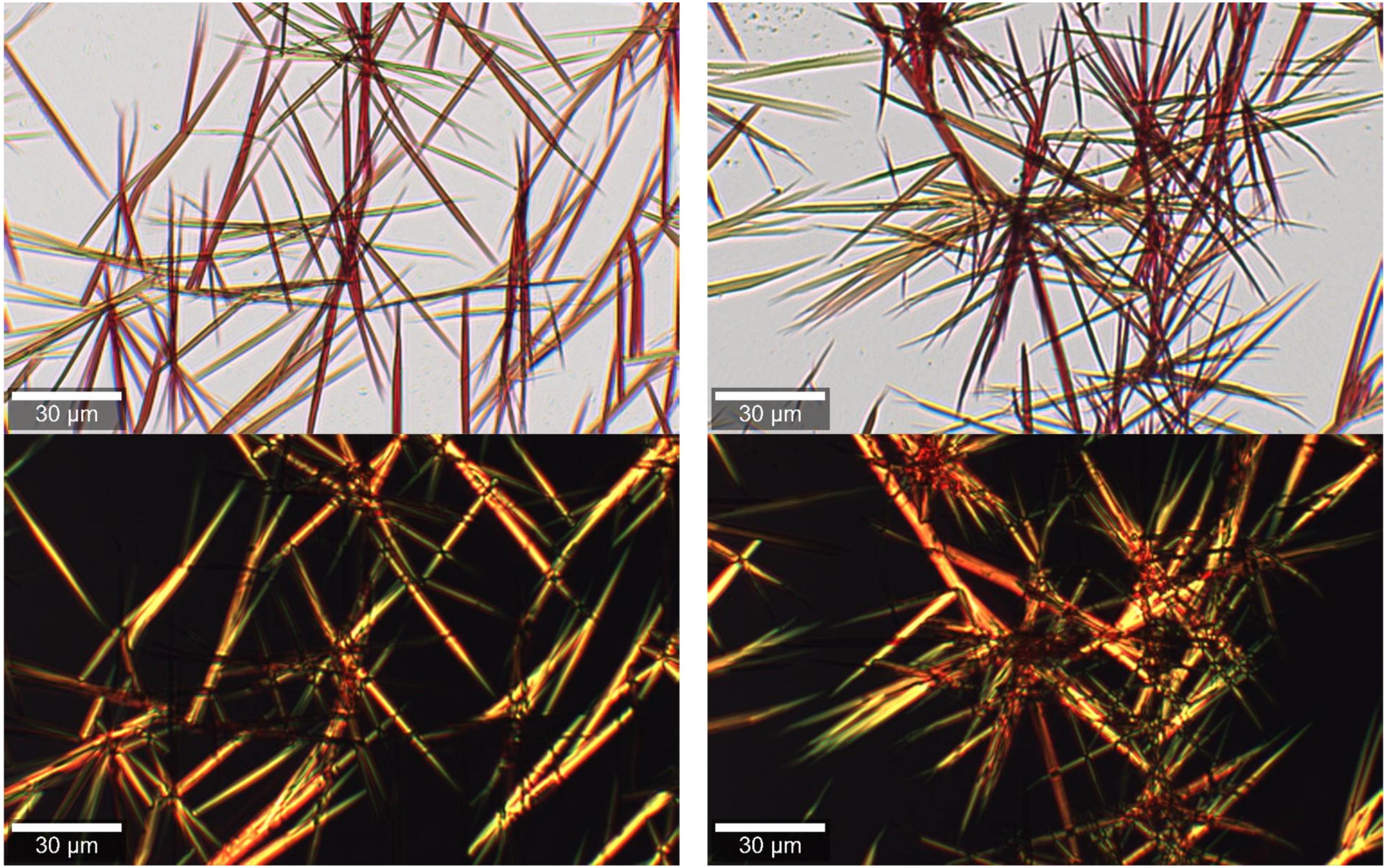

Fig. S22. Representative brightfield and polarization images of **3** formed from MeOH solutions.

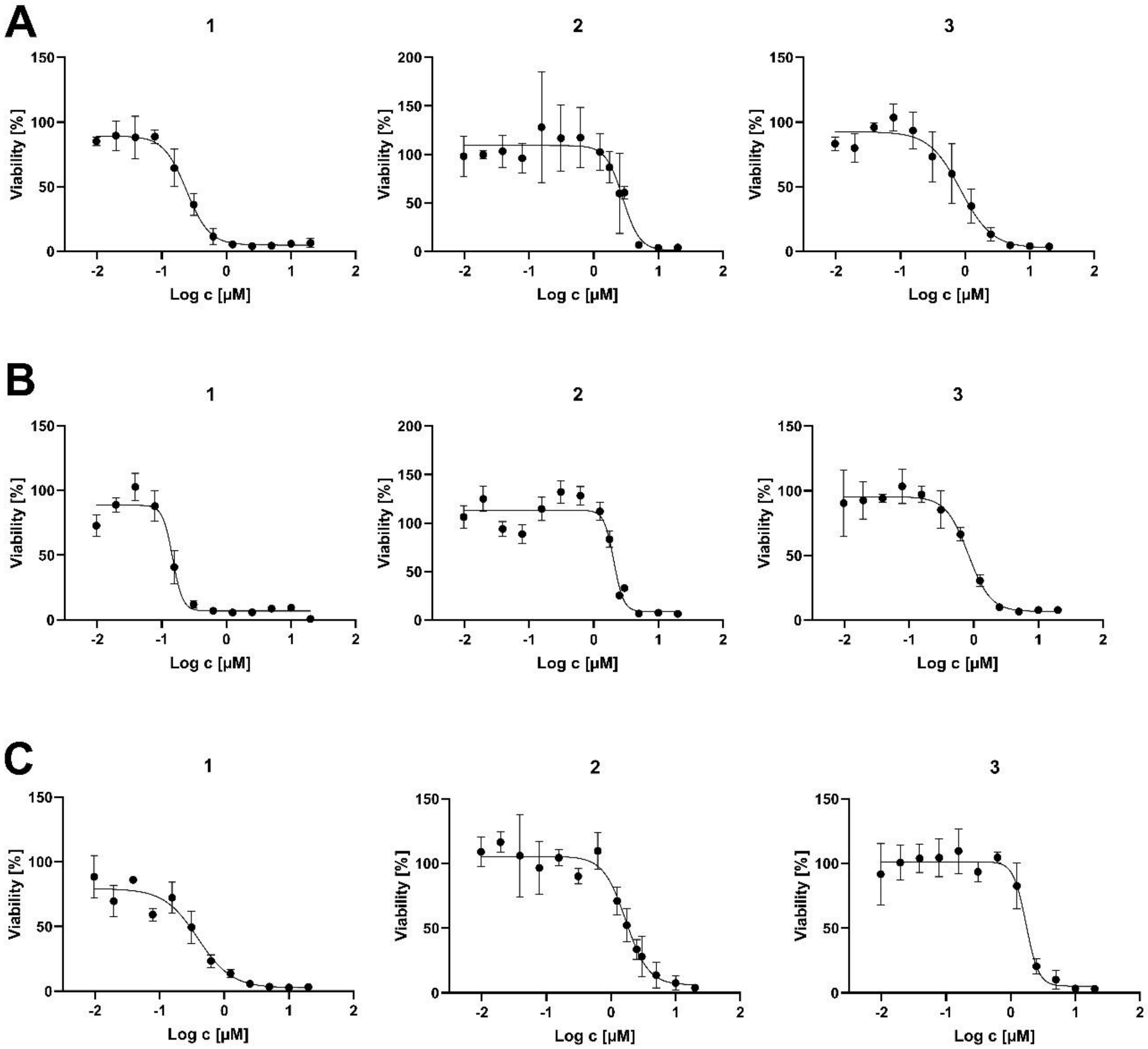

Fig. S23. Dose-dependent viability of A) HCT116, B) MDA-MB-231, and C) hTERT-RPE-1 cell lines treated by **1**–**3**.

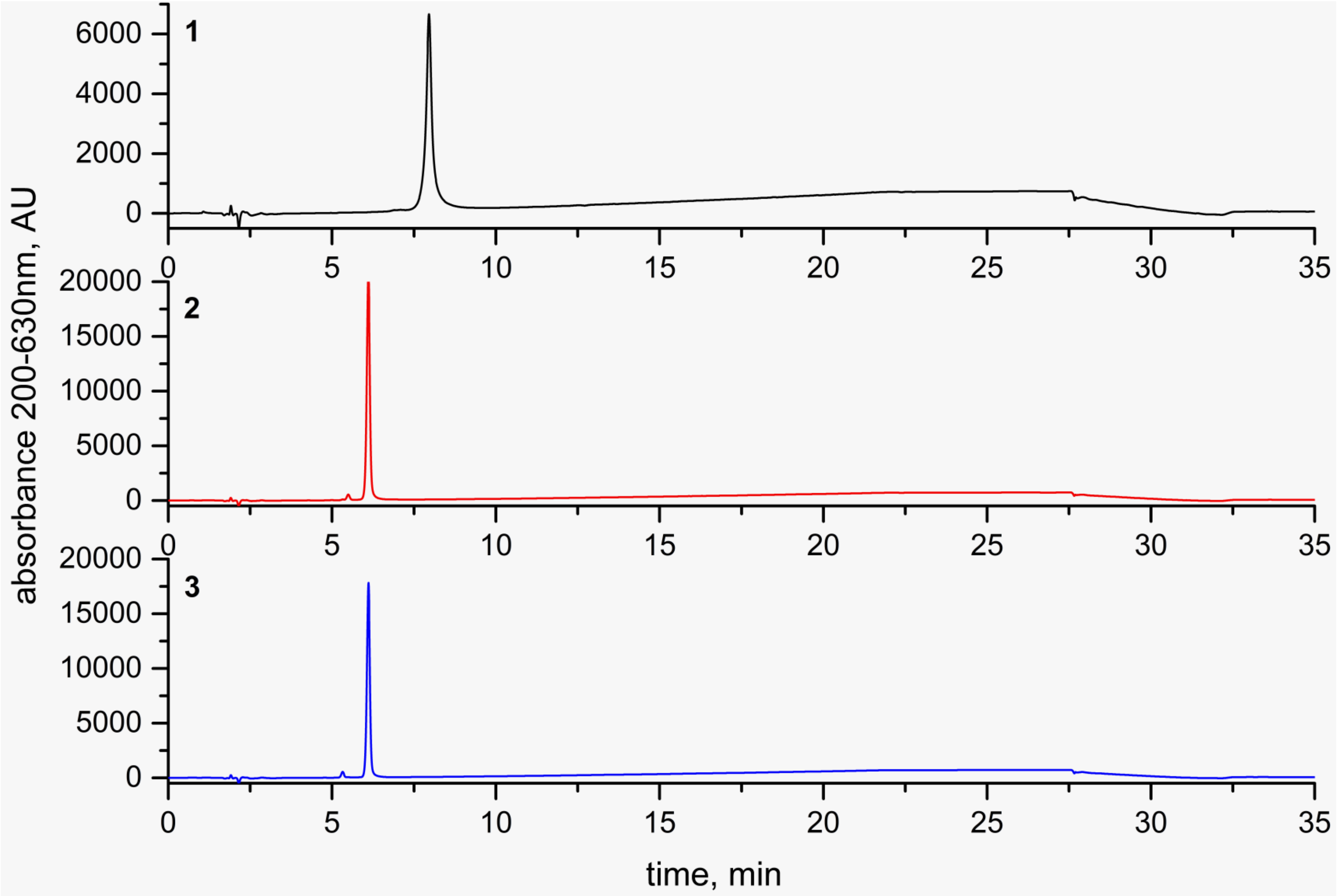

Fig. S24. HPLC-DAD chromatogram of **1–3**.

Table S1. List of detected correlation for Compound **1**

Atom	δ (ppm)	COSY	HSQC	HMBC
C-1	171.4			H-7
C-2	140.8			H-15[a]
C-3	140.3			H-14[a]
C-4	172.3			
C-5	167.34			H-7, H-11, H-14
C-6	148.8			H-11, H-12, H-14
C-7	130.9			H-11, H-15
H-7	7.08			C-1, C-5, C-9, C-11
C-8	167.6			H-15
C-9	109.5			H-7, H-15
C-10	110.2			H-14
C-11	32.0		H-11	H-7, H-12, H-13
H-11	2.68	H-12	C-11	C-5, C-6, C-7, C-12, C-13
C-12	21.9		H-12	H-11, H-13
H-12	1.68	H-11, H-13	C-12	C-6, C-11, C-13
C-13	14.00		H-13	H-11, H-12
H-13	1.01	H-12	C-13	C-11, C-12
H-14	12.95			C-3, C-5, C-6, C-10
H-15	12.60			C-2, C-7, C-8, C-9

[a] This very weak correlation can be explained by fast exchange between tautomers 5,8-dioxo-1,4-dihydroxo, and 5,8-dihydroxo-1,4-dioxo analogical to tautomerism described in Carreño et al.[1] (see SI REFERENCES).

Table S2. Selected natural NQ products structurally related to **1–3** For [2-12] see SI REFERENCES

**Compound**	**Deoxylapachol[2,3]**	**3-ethyl-2,7-dihydroxy-naphthazarin[4]**	**Cristazarin[5]**	**Droserone[6]**	**Plumbagin[7]**	**Chloroplumbagin[7,8]**
**Structure**	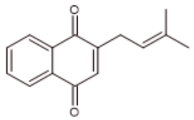	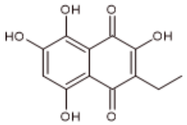	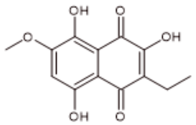	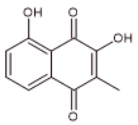	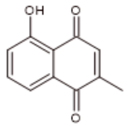	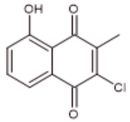
**Chemical formula**	$C_{15}H_{14}O_2$	$C_{12}H_{10}O_6$	$C_{13}H_{12}O_6$	$C_{11}H_8O_4$	$C_{11}H_8O_3$	$C_{11}H_7ClO_3$
**Occurrence**	Brown alga *Landsburgia quercifolia*; heartwood of *Tabebuia* sp.	Lichen *Cetraria laevigata*	Lichen *Cladonia cristatella* – mycobiont	Drosera, Sundews, Diospyros	Plumbago, Sundews	Drosera, Sundews, Diospyros
**Biological Effects**	Cytotoxic, antitumour	Antioxidant, cytotoxic	Antibacterial, antitumour, cytotoxic	Antifungal, antitussive (against cough)	Antitumor, detoxification, antiviral, antibacterial, hypolipidemic	Cytotoxic, detoxification, antitumor

Table S2. Selected natural NQ products structurally related to **1–3** – continued

**Juglone**[9,10]	**Lawsone**[11]	**Echinochrome A**[12]	**Spinochrome E**[13]
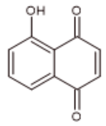	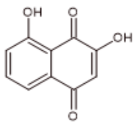	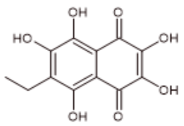	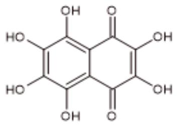
$C_{10}H_6O_3$	$C_{10}H_6O_4$	$C_{12}H_{10}O_7$	$C_{10}H_6O_8$
Walnuts (Juglans)	Henna (Lawsonia)	Sea urchins	Sea urchins
Allelopathic, antitumor, antibacterial, antiviral, prooxidation	Dyeing, oxidative stress	Antioxidant, antiviral	Antioxidant

SI REFERENCES

(1) Carreño, C. M.; García Ruano, L. J.; Urbano, A. Tautomeric Equilibrium of Naphthazarin Thioderivatives. *Tetrahedron* **1994**, *50* (17), 5013–5020. https://doi.org/10.1016/S0040-4020(01)90412-0.

(2) Perry, N. B.; Blunt, J. W.; Munro, M. H. G. A Cytotoxic and Antifungal 1,4-Naphthoquinone and Related Compounds from a New Zealand Brown Alga, Landsburgia Quercifolia. *J. Nat. Prod.* **1991**, *54* (4), 978–985. https://doi.org/10.1021/np50076a009.

(3) Sunassee, S. N.; Veale, C. G. L.; Shunmoogam-Gounden, N.; Osoniyi, O.; Hendricks, D. T.; Caira, M. R.; De La Mare, J. A.; Edkins, A. L.; Pinto, A. V.; Da Silva Júnior, E. N.; Davies-Coleman, M. T. Cytotoxicity of Lapachol, β-Lapachone and Related Synthetic 1,4-Naphthoquinones against Oesophageal Cancer Cells. *Eur. J. Med. Chem.* **2013**, *62* (5), 98–110. https://doi.org/10.1016/j.ejmech.2012.12.048.

(4) Prokopiev, I.; Sleptsov, I.; Serebryakov, E.; Sharoyko, V. Antioxidant and Cytotoxic Activities of Quinones from *Cetraria Laevigata*. *Nat. Prod. Res.* **2024**, *38* (4), 685–689. https://doi.org/10.1080/14786419.2023.2187795.

(5) Jeong, M.-H.; Park, C.-H.; Kim, J. A.; Choi, E. D.; Kim, S.; Hur, J.-S.; Park, S.-Y. Production and Activity of Cristazarin in the Lichen-Forming Fungus Cladonia Metacorallifera. *Journal of Fungi* **2021**, *7* (8), 601. https://doi.org/10.3390/jof7080601.

(6) Thomson, R. H. Naphthoquinones. In *Naturally Occurring Quinones IV: Recent advances*; Springer Netherlands: Dordrecht, 1997; pp 112–308. https://doi.org/10.1007/978-94-009-1551-0_2.

(7) Tanwar, A. K.; Chatterjee, D.; Jain, N.; Sharma, S.; Tikoo, K.; Singh, I. P. Chemical Basis of the Traditional Ayurvedic Detoxification Process of the Toxic Medicinal Plant *Plumbago Zeylanica*. *J. Nat. Prod.* **2025**, *88* (1), 15–23. https://doi.org/10.1021/acs.jnatprod.3c00975.

(8) Kawiak, A.; Domachowska, A.; Krolicka, A.; Smolarska, M.; Lojkowska, E. 3-Chloroplumbagin Induces Cell Death in Breast Cancer Cells Through MAPK-Mediated Mcl-1 Inhibition. *Front. Pharmacol.* **2019**, *10*, 784. https://doi.org/10.3389/fphar.2019.00784.

(9) Meyer, G. W.; Naranjo, M. A. B.; Widhalm, J. R. Convergent Evolution of Plant Specialized 1,4-Naphthoquinones: Metabolism, Trafficking, and Resistance to Their Allelopathic Effects. *J. Exp. Bot.* **2021**, *72* (2), 167–176. https://doi.org/10.1093/jxb/eraa462.

(10) Tang, Y. T.; Li, Y.; Chu, P.; Ma, X. D.; Tang, Z. Y.; Sun, Z. L. Molecular Biological Mechanism of Action in Cancer Therapies: Juglone and Its Derivatives, the Future of Development. *Biomedicine & Pharmacotherapy* **2022**, *148*, 112785. https://doi.org/10.1016/j.biopha.2022.112785.

(11) Xavier MR; Santos MMS; Queiroz MG; de Lima Silva MS; Goes AJS; De Morais MA. Lawsone, a 2-Hydroxy-1,4-Naphthoquinone from Lawsonia Inermis (Henna), Produces Mitochondrial Dysfunctions and Triggers Mitophagy in Saccharomyces Cerevisiae. *Mol. Biol. Rep.* **2020**, *47* (11), 1173–1185.

(12) Jeong, S.; Kim, H.; Song, I.-S.; Lee, S.; Ko, K.; Rhee, B.; Kim, N.; Mishchenko, N.; Fedoryev, S.; Stonik, V.; Han, J. Echinochrome A Protects Mitochondrial Function in Cardiomyocytes against Cardiotoxic Drugs. *Mar. Drugs* **2014**, *12* (5), 2922–2936. https://doi.org/10.3390/md12052922.

(13) Zhou, D. Y.; Qin, L.; Zhu, B. W.; Wang, X. D.; Tan, H.; Yang, J. F.; Li, D. M.; Dong, X. P.; Wu, H. T.; Sun, L. M.; Li, X. L.; Murata, Y. Extraction and Antioxidant Property of Polyhydroxylated Naphthoquinone Pigments from Spines of Purple Sea Urchin Strongylocentrotus Nudus. *Food Chem.* **2011**, *129* (4), 1591–1597. https://doi.org/10.1016/j.foodchem.2011.06.014.
